# Transcription factors, sucrose, and sucrose metabolic genes interact to regulate potato phenylpropanoid metabolism

**DOI:** 10.1093/jxb/ert303

**Published:** 2013-10-05

**Authors:** Raja S. Payyavula, Rajesh K. Singh, Duroy A. Navarre

**Affiliations:** ^1^Irrigated Agricultural Research and Extension Center, Washington State; University, Prosser, WA 99164, USA; ^2^Department of Horticulture and Landscape Architecture, Washington State University, Pullman, WA 99164, USA; ^3^USDA-Agricultural Research Service 24106 North Bunn Road, Prosser, WA 99350, USA

**Keywords:** Anthocyanins, bHLH, chlorogenic acid, nutrition, MYB, phenolics, phenylpropanoids, phytonutrients, potato, sucrose, tobacco.

## Abstract

Much remains unknown about how transcription factors and sugars regulate phenylpropanoid metabolism in tuber crops like potato (*Solanum tuberosum*). Based on phylogeny and protein similarity to known regulators of phenylpropanoid metabolism, 15 transcription factors were selected and their expression was compared in white, yellow, red, and purple genotypes with contrasting phenolic and anthocyanin profiles. Red and purple genotypes had increased phenylalanine ammonia lyase (PAL) enzyme activity, markedly higher levels of phenylpropanoids, and elevated expression of most phenylpropanoid structural genes, including a novel anthocyanin *O*-methyltransferase. The transcription factors *Anthocyanin1* (*StAN1*), *basic Helix Loop Helix1* (*StbHLH1*), and *StWD40* were more strongly expressed in red and purple potatoes. Expression of 12 other transcription factors was not associated with phenylpropanoid content, except for *StMYB12B*, which showed a negative relationship. Increased expression of *AN1*, *bHLH1*, and *WD40* was also associated with environmentally mediated increases in tuber phenylpropanoids. Treatment of potato plantlets with sucrose induced hydroxycinnamic acids, flavonols, anthocyanins, structural genes, *AN1*, *bHLH1*, *WD40*, and genes encoding the sucrose-hydrolysing enzymes SUSY1, SUSY4, and INV2. Transient expression of *StAN1* in tobacco leaves induced *bHLH1*, structural genes, *SUSY1*, *SUSY4*, and *INV1*, and increased phenylpropanoid amounts. *StAN1* infiltration into tobacco leaves decreased sucrose and glucose concentrations. *In silico* promoter analysis revealed the presence of MYB and bHLH regulatory elements on sucrolytic gene promoters and sucrose-responsive elements on the *AN1* promoter. These findings reveal an interesting dynamic between *AN1*, sucrose, and sucrose metabolic genes in modulating potato phenylpropanoids.

## Introduction

**Fig. 1.**
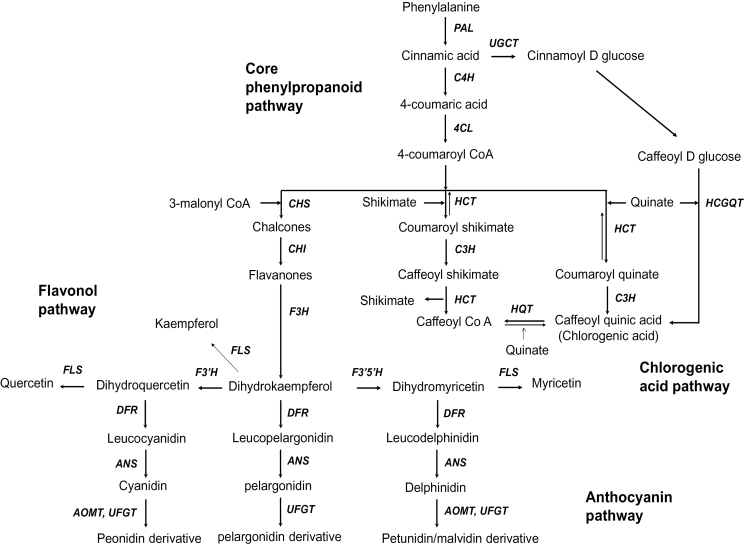
The plant phenylpropanoid pathway. PAL, phenylalanine ammonia lyase; C4H, cinnamate 4-hydroxylase; 4CL, 4-coumaroyl:CoA-ligase; C3H, *p*-coumarate 3-hydroxylase; HCT, hydroxycinnamoyl-CoA shikimate hydroxycinnamoyl transferase; HQT, hydroxyl-cinnamoyl CoA quinate hydroxycinnamoyl transferase; UGCT, UDP-glucose:cinnamate glucosyl transferase; HCGQT, hydroxycinnamoyl glucose:quinate hydroxycinnamoyl transferase, CHS, chalcone synthase; CHI, chalcone isomerase; F3H, flavanone 3-hydroxylase; F3′H, flavonoid 3′ hydroxylase; F3′5′H, flavonoid 3′,5′-hydroxylase; FLS, flavonol synthase; DFR, dihydroflavonol 4-reductase; ANS, anthocyanin synthase; UFGT, UDPG flavonoid *O*-glucosyltransferase; AOMT, anthocyanin methyltransferase.

Plants synthesize an array of phenylpropanoids with diverse roles including in plant growth and development, flowering, pigmentation, signal transduction, and structural integrity (Fig. 1; Koes *et al.*, 2005; Vogt, 2010). Phenylpropanoids are also important plant dietary constituents that possess various health-promoting properties, including against cardiovascular disease and cancers (Parr and Bolwell, 2000). White potatoes (*Solanum tuberosum L.*) contain modest amounts of phenylpropanoids and are the third largest source of dietary phenylpropanoids because of high consumption (Chun *et al.*, 2005). Red- and purple-flesh potatoes contain decidedly higher amounts of phenylpropanoids (André *et al.*, 2007a) and the increase is largely, but not solely, due to anthocyanin biosynthesis (Navarre *et al.*, 2011).

**Fig. 2.**
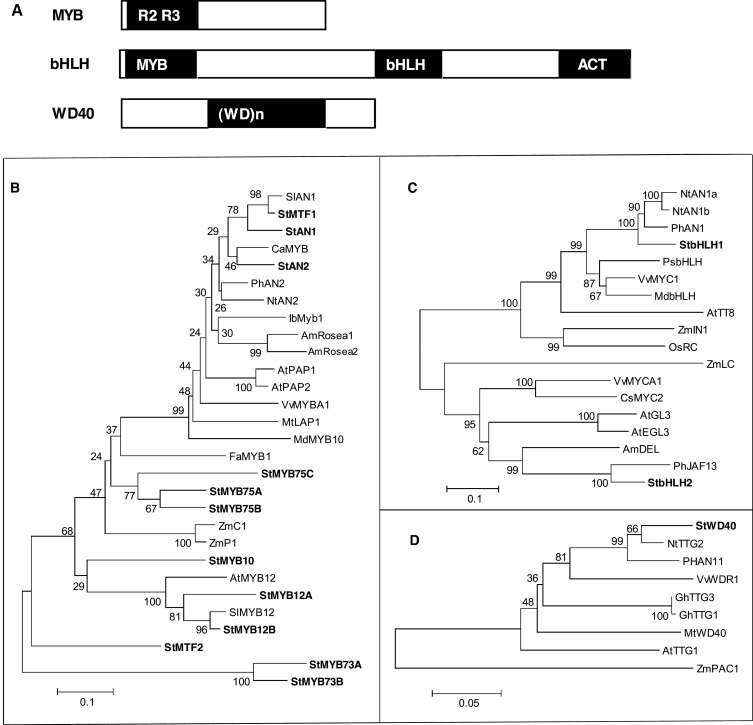
(A) Structures of MYB, basic helix–loop–helix (bHLH), and WD40 proteins. (B–D) Phylogenetic trees of transcription factors from potato and other species: MYB (B), bHLH (C), and WD40 (D). Proteins from potato are in bold. The evolutionary history was inferred using the neighbour-joining method. NCBI Protein and Swiss-Prot accession numbers: AmRosea1 (*Antirrhinum majus*): ABB83826; AmRosea2: ABB83827; AtPAP1 (*Arabidopsis thaliana*): AAG42001; AtPAP2: AAG42002; AtMYB12: NP_182268; CaMYB (*Capsicum annuum*): CAE75745; FaMYB1 (*Fragaria ananassa*):AAK84064; IbMYB1 (*Ipomoea batatas*): BAF45114; MdMyb10 (*Malus domestica*): ABB84753; MtLAP1 (*Medicago truncatula*): ACN79541; NtAn2 (*Nicotiana tabacum*): ACO52470; OsMyb4 (*Oryza sativa*): T02988; PhAN2 (*Petunia hybrida*): AAF66727; SlAN1 (*Solanum lycopersicum*): AAQ55181; SlMYB12: ACB46530; StAn1 (*Solanum tuberosum*): PGSC0003DMT400036281; StAn2, PGSC0003DMT400036284; StMTF1: ABY40370; MYB73A: PGSC0003DMT400064306; MYB73B PGSC0003DMT400008569; StMYB75A: PGSC0003DMT400014983; StMYB75B: PGSC0003DMT400031607; StMYB75C: PGSC0003DMT400085829; StMYB12A: PGSC0003DMT400018841; StMYB12B: PGSC0003DMT400023322; StMTF2: PGSC0003DMT400017844; StMYB10: PGSC0003DMT400060168; VvMYBA1 (*Vitis vinifera*): BAD18977; ZmC1 (*Zea mays*): AAA33482; ZmPl: AAA19819; AmDEL: AAA32663; AtGL3: NP_680372; AtEGL3: NP_176552; AtTT8: CAC14865; CsMYC2 (*Citus sinensis*): ABR68793; MdbHLH: ADL36597; NtAn1a: AEE99257; NtAn1b, AEE99258; OsRC: BAF42668; PhAn1: AAG25927; PhJAF13: AAC39455; PsbHLH, (*Pisum sativum*): ADO13282; StbHLH1, PGSC0003DMT400033569; StbHLH2, PGSC0003DMT400032139; VvMYCA1: ABM92332; VvMYC1: ACC68685; ZmIN1, AAB03841; ZmLc, NP_001105339; AtTTG1:CAC10524; GhTTG1 (*Gossypium hirsutum*): AAK19614; GhTTG3: AM95645; MtWD40: ABW08112; NtTTG2: ACN87316; PhAN11: AAC18914; VvWDR1: ABF66625; ZmPAC1: AAM76742.

Numerous factors mediate expression of phenylpropanoid genes, including sugars and various transcription factors including MYBs (Dubos *et al.*, 2010). MYBs have single or multiple imperfect repeats (R) of structurally conserved DNA-binding domains (Fig. 2A). R2R3 genes comprise the largest of the four classes of plant MYBs and have a conserved N-terminal DNA-binding domain that is in direct contact with the DNA and a variable C-terminal domain that activates or represses its targets (Dubos *et al.*, 2010). R2R3 MYBs are divided into 22 subgroups in *Arabidopsis thaliana* based on conserved motifs (Stracke *et al.*, 2001). Various R2R3 MYBs regulate phenylpropanoid biosynthesis, some of which interact with basic helix–loop–helix (bHLH) proteins (Grotewold, 2005). A mutant maize (*Zea mays*) P1 MYB activates some flavonoid genes but not anthocyanins in the absence of a bHLH, whereas AtMYB12 regulates flavonol biosynthesis without a bHLH interaction (Grotewold *et al.*, 2000; Mehrtens *et al.*, 2005).

Unlike the flavonol branch of the pathway, anthocyanin biosynthesis is typically regulated by a complex in which MYB, bHLH, and WD40 transcription factors interact. When this complex is formed, MYB and bHLH bind to promoters with consensus nucleotide sequences like MACCWAMC and CANNTG (Sablowski *et al.*, 1994; Zimmermann *et al.*, 2004). The first 200 aa of bHLH proteins are required to interact with MYB transcription factors, and aa 200–400 interact with WD40 proteins (Pattanaik *et al.*, 2010). The C-terminal ACT-like domain facilitates binding of MYB to the promoter (Grant, 2006). WD proteins have four to eight imperfect tandem repeats and interact with other proteins through the WD repeat region (Neer *et al.*, 1994).

Sucrose modulates transcriptional and post-translational regulation of many pigment-related genes (Koch, 1996). Sucrose induces anthocyanins in *Arabidopsis* through induction of *PAP1/MYB75* (Production of Anthocyanin Pigment 1) and fails to induce anthocyanins in the *pap1* mutant (Teng *et al.*, 2005; Solfanelli *et al.*, 2006). Anthocyanin induction in *Arabidopsis* seems specific to sucrose, but in grapes (*Vitis vinifera*), other sugars also stimulate anthocyanin synthesis (Gollop *et al.*, 2002).

As a staple food, potatoes are an attractive target for phytonutrient enhancement. Tuber-specific overexpression of the MYB transcription factor *StMtf1* resulted in elevated amounts of phenylpropanoids (Rommens *et al.*, 2008). Tuber anthocyanin synthesis in the periderm is controlled in part by three loci, *D*, *P*, and *R*. *P* and *R* were found to be structural genes, whereas *D* encodes an R2R3 MYB (Jung *et al.*, 2005, 2009; Zhang *et al.*, 2009a). The *D* locus maps to a region of chromosome 10 that harbours *StAN2*, which was later renamed *StAN1* (Jung *et al.*, 2009). *StAN1* expression correlated with anthocyanin levels in drought-stressed potatoes (André *et al.*, 2009).

The goal of this study was to determine how sugars and transcription factors modulate biosynthesis of potato phenylpropanoids, including hydroxycinnamic acids, flavonols, and anthocyanins. The involvement of sugars and 15 transcription factors was characterized and provided evidence that AN1 along with bHLH1, sucrose, and sucrose metabolic genes interact to regulate the pathway.

## Materials and methods

### Plant materials

Small tubers (~25–50g) from NY144, Challenger, ORO4198-1, AmaRosa and Magic Molly were harvested from field-grown plants in Moses Lake, WA, USA, in August 2011. Tubers were peeled and frozen in liquid nitrogen within minutes of harvest. Analysis of environmental influences on transcription factor expression was performed on potatoes grown in Texas, Florida, and four locations in Alaska (Payyavula *et al.*, 2012). For sucrose feeding studies, internodes from 1-month-old potato (cv. Purple Majesty) plants were propagated on MS medium supplemented with 0 or 120mM sucrose at 25 °C with 16h light. Plantlets were collected after 5 d.

### Phenylpropanoid analysis

Phenolics were extracted from 50mg freeze-dried homogenized sample with 1.5ml of 50% methanol, 1mM EDTA, and 2.5% metaphosphoric acid using a validated method (Shakya and Navarre, 2006). Total phenolics were estimated by the Folin–Ciocalteu method (Singleton and Rossi, 1965). Individual phenolics were analysed on a 100×4.6mm Onyx monolithic C-18 (Phenomenex) column with an Agilent 1100 HPLC system equipped with a quaternary pump, refrigerated autosampler, and column heater. Detectors were a DAD and SL ion trap with an electrospray ionization (ESI) source operated in both positive and negative ion mode, as described previously (Navarre *et al.*, 2011). Anthocyanins were extracted from 50mg of dry powder using a total of 2ml of 50% methanol containing 2.5% formic acid. Total anthocyanins were estimated by a pH differential method (Wrolstad *et al.*, 2005). Individual anthocyanins were determined by liquid chromatography/ mass spectrometry (LC/MS) as described previously (Payyavula *et al.*, 2012). Retention times and MS data for the quantitated compounds are shown in Supplementary Tables S1 and S2 at *JXB* online.

### RNA extraction, cDNA synthesis, and quantitative real-time PCR (qRT-PCR)

RNA was extracted using hot cetyl trimethylammonium bromide (Chang *et al.*, 1993), with slight modification (Payyavula *et al.*, 2012). Samples were quantified with a NanoDrop ND-1000 spectrophotometer (NanoDrop Technologies) and quality was assayed on a 1% agarose gel. cDNA synthesis used 2 μg of total RNA, Moloney murine leukemia virus reverse transcriptase (New England BioLabs), and oligo(dT) 20VN primers at 42 °C for 2h. Quantitative PCR was performed in 384-well plates on a LightCycler 480 (Roche) with 3.5ng of equivalent cDNA. Relative abundance was calculated with the Δ*C*_T_ method (Livak and Schmittgen, 2001) using elongation factor-1, actin, and ribosomal protein L2 for template normalization. The primers are listed in Supplementary Table S3 at *JXB* online. Statistical analysis was performed with standard analysis of variance using SAS 9.2 Proc GLM. The means were obtained using LSMEANS with Tukey adjustments for the degrees of freedom and *P*<0.05 default separation (Rourke *et al.*, 2005). Pearson correlation coefficients were calculated using Microsoft Excel and visualized with HeatMapper Plus (Verhaak *et al.*, 2006).

### Phylogenetic tree and protein similarity

Protein sequences collected by the best BLAST match from the Potato Genome Sequencing Consortium (PGSC) database (http://solanaceae.plantbiology.msu.edu/pgsc_download.shtml) or NCBI were aligned and a phylogenetic tree developed using MEGA4 (Tamura *et al.*, 2007). The evolutionary history was inferred using the neighbour-joining method with 500 bootstrap replicates. The evolutionary distances were computed using the Poisson correction method with units of the number of amino acid substitutions per site. Percentage similarity was calculated with GeneDoc (Nicholas *et al.*, 1997).

### Antioxidant and enzyme assays

A ferric reducing antioxidant power (FRAP) assay was performed as reported elsewhere (Benzie and Strain, 1996). To an aliquot of 15–30 μl of phenolic extract, 1ml of the pre-warmed (37 °C) FRAP reagent was added, incubated 5min at 37 °C, and then centrifuged for 1min. Absorbance at 593nm (*A*_593_) was read and antioxidant capacity was estimated as trolox equivalents. Phenylalanine ammonia lyase (PAL) catalyses the first reaction of the phenylpropanoid pathway (Fig. 1) and activity was measured as described previously (Zucker, 1965). In a 1.5ml tube, 700 μl of 50mM sodium borate buffer (pH 8.8) and 200 μl of 50mM phenylalanine were pre-warmed at 37 °C prior to adding 100 μl of extract. The reaction was continued for 1h at 37 °C and then stopped with 200 μl of 12% trichloroacetic acid and measured at *A*_290_. Sugars were extracted twice at 80 °C for 15min from 25mg of freeze-dried sample with a total of 2ml of 80% ethanol. Pigments were removed by re-extracting with 50mg of activated charcoal. Sucrose and glucose were estimated using Sigma kits (SCA20 and GAHK20).

### Cloning and leaf infiltrations

Full-length coding sequences of the potato transcription factors AN1, bHLH1, and WD40 and the structural gene AOMT3 (anthocyanin *O*-methyltransferase 3) were amplified from Magic Molly or Purple Majesty tuber cDNA using Phusion^®^ DNA polymerase (New England Biolabs). Full-length fragments were ligated into the modified binary vector pOREO2 with a 35S promoter (Coutu *et al.*, 2007). Clones were confirmed by sequencing (Retrogen). Sequences for StAN1 (JX848659), StbHLH1 (JX848660), StWD40 (JX848661) and StAOMT3 (JX848662) were submitted to GenBank.

A single positive colony was cultured in 5ml of LB medium overnight and used to inoculate 50ml of medium. Cells were harvested and redissolved in 10mM MgCl_2_ containing 100 μM acetosyringone and adjusted to an optical density of 0.5. Cultures were then diluted (1:1) with the gene silencing suppression vector p19^TBSV^ of tomato bushy stunt virus (Voinnet *et al.*, 2003) to avoid co-suppression. Samples were infiltrated into leaves of 3-week-old tobacco plants (*Nicotiana tabacum* cv. Samsun and *Nicotiana benthamiana*) maintained in a growth chamber under conditions of 15h light. Leaves were harvested at 4 d post-infiltration.

## Results

### Phylogenetic analysis and protein similarity

A total of 12 MYBs were selected for characterization based on BLAST searches conducted against the potato genome database (The Potato Genome Sequencing Consortium, 2011) using nucleotide sequences of transcription factors described previously in the literature. *Arabidopsis* has 198 MYB genes (Yanhui *et al.*, 2006) of which *AtPAP1* (GenBank accession no. AF325123) and *AtPAP2* (MYB90, AF325124) are known to be involved in anthocyanin synthesis (Dubos *et al.*, 2010). Three putative potato MYB genes, *StMYB75A*, *StMYB7B*, and *StMYB75C*, were identified based on homology to *AtPAP1* and *AtPAP2*. *StMYB12A* and *StMYB12B* are homologous to *SlMYB12* (EU419748) and *AtMYB12* (NM_130314), which regulate flavonols in tomato (*Solanum lycopersicum*) and *Arabidopsis* (Mehrtens *et al.*, 2005). *StAN1* and *StAN2* (AY841127 and AY841131) are MYBs implicated in regulating anthocyanin biosynthesis in tuber skin and drought-stressed potatoes (André *et al.*, 2009; Jung *et al.*, 2009). Numerous sequences were found in the PGSC database with homology to *StAN1* and *StAN2*, but only the two with highest similarity were chosen. *StMTF1* (EU310399) and *StMTF2* (CV506186) are MYBs shown to regulate potato phenylpropanoids with varying efficacy (Rommens *et al.*, 2008). *StMYb73A* and *StMYb73B* were collected by blasting a *MYB73* sequence that was upregulated 44-fold in the purple portions of tubers compared with the white portions (Stushnoff *et al.*, 2010). *StMYB10* was homologous to *NtAn2* (FJ472647) and *MdMYB10* (EU518249), which regulate anthocyanin biosynthesis in tobacco floral tissue and apple (*Malus domestica*), respectively (Espley *et al.*, 2007; Pattanaik *et al.*, 2010).

*AtTT8* (AJ277509), *AtGL3* (NM_148067.3), *JAF13* (AF020545.1), and *NtAN1* (HQ589209) are *bHLH* genes involved in anthocyanin synthesis (Nesi *et al.*, 2000; Quattrocchio *et al.*, 1998; Feyissa *et al.*, 2009; Bai *et al.*, 2011). After BLASTing these sequences, the two best matches in potato were *bHLH1* and *bHLH2*. *AtTTG1* (AJ133743), *MtWD40-1* (EU040206), and *PhAN11* (U94748) are *WD40* members that regulate anthocyanin synthesis (Vetten *et al.*, 1997; Walker *et al.*, 1999; Pang *et al.*, 2009) and their sequences were used to identify *StWD40*.

The protein sequences of these 12 MYBs, two bHLHs, and one WD40 from potato were used to develop a phylogenetic tree (Fig. 2B–D). StAN1 and StAN2 were closely associated with the MYBs of other solanaceous species, SlAN1, NtAN2, and CaMYB, known to regulate anthocyanins (Mathews *et al.*, 2003; Borovsky *et al.*, 2004; Pattanaik *et al.*, 2010). StAN1 was 66 and 58% similar to StAN2 and NtAN2, respectively, and StMTF1 was 89% similar to SlAN1 (Supplementary Table S4A at *JXB* online). StMYB12A and StMYB12B clustered with AtMYB12 and SlMYB12 (Luo *et al.*, 2008; Ballester *et al.*, 2010). MYB73A and MYB73B formed a unique clade. All the MYBs from solanaceous species had highly conserved R2 and R3 MYB domains (Supplementary Fig. S1A at *JXB* online). StbHLH1 clustered and shared around 80% similarity with NtAN1a and PhAN1, which regulate anthocyanins in tobacco and petunia (*Petunia hybrida*) and was only 43% similar to StbHLH2. However, StbHLH2 was 86% similar to PhJAF13 (Supplementary Table S4B). These proteins are conserved in MYB and bHLH domains but are diversified in other regions (Supplementary Fig. S1B).

StWD40 is clustered with NtTTG2, PhAN11, and VvWDR1, and is 97% similar to NtTTG2 and 94 and 88% similar to PhAN11 and VvWDR1, respectively (Supplementary Table S4C), which regulate anthocyanin synthesis in petunia and grapes (Vetten *et al.*, 1997; Matus *et al.*, 2010). These proteins are slightly distinct at the N-terminal end but are highly conserved in the middle and C-terminal end (Supplementary Fig. S1C).

### Basal phenylpropanoid metabolism

**Fig. 3.**
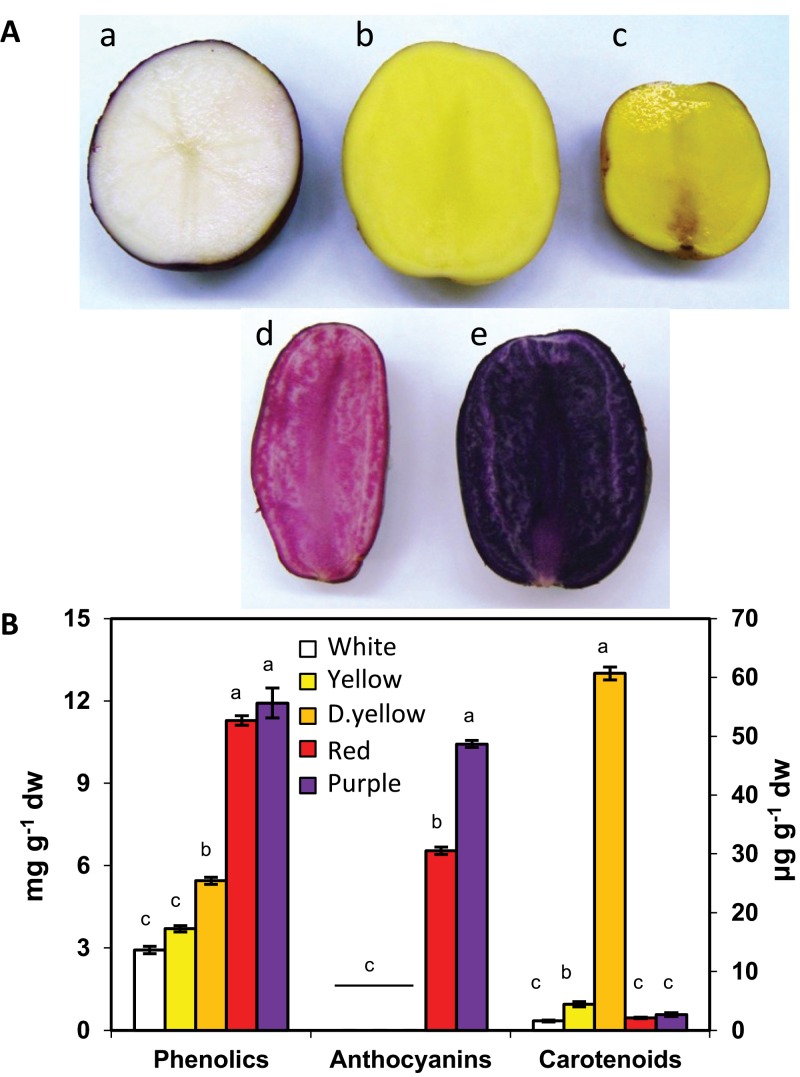
Characterization of the five genotypes used in this study. (A) Flesh colour of NY144 (a), Challenger (b), ORO4198-1 (c), AmaRosa (d), and Magic Molly (e). (B) Levels of total phenolics, anthocyanins, and carotenoids. Carotenoids are shown on the *y*-axis on the right. The data represents the means±SEM of three biological replicates. Values with the same letter are not significantly different (*P*<0.05).

Five genotypes, NY144, Challenger, ORO4198-1, AmaRosa and Magic Molly with white, light yellow, dark yellow, red, and purple flesh, respectively, were selected for analysis (Fig. 3A). These genotypes were chosen because they were expected to have markedly different phenylpropanoid profiles; consequently, a comparative analysis of transcription factor expression would be informative. Prior to transcription factor analysis and to provide a context to interpret the results, phenylpropanoid profiles and structural gene expression were evaluated in each genotype. Tubers were collected from field-grown plants, peeled, and processed within minutes of harvest to avoid potential post-harvest effects. Total phenolic levels ranged from 2.9 to 12mg g^–1^ (Fig. 3B) and were 3–4-fold higher in red and purple potatoes than in white. Anthocyanins were detectable only in red and purple cultivars, with higher amounts in the purple (10.4mg g^–1^) than in the red (6.5mg g^–1^) genotype. Carotenoids were highest (60.7 μg g^–1^) in the dark yellow genotype.

**Fig. 4.**
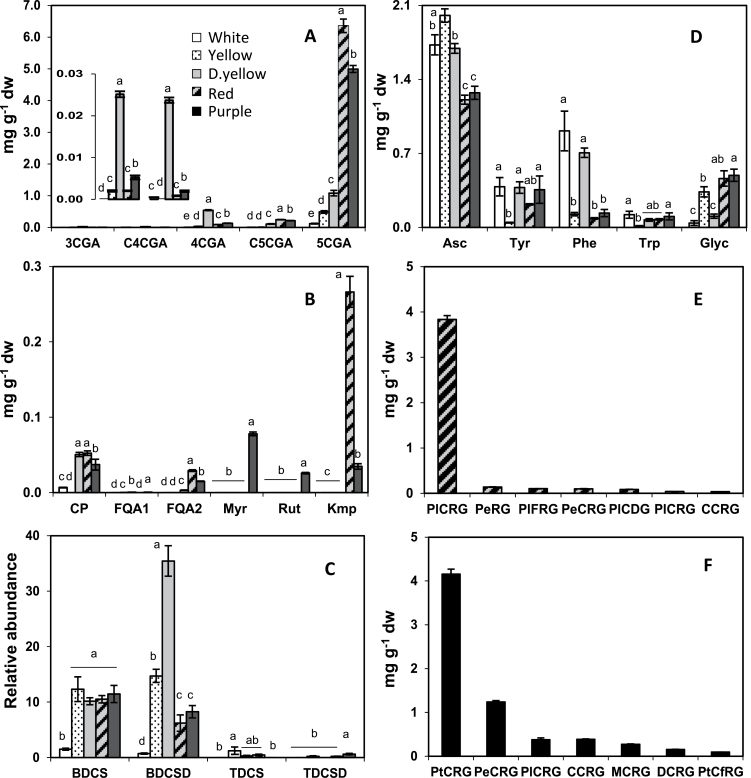
Individual metabolites measured by LC/MS in the five genotypes. 5CGA and CGA isomers, with 3CGA and C4CGA in the inset (A); caffeoyl and feruloyl derivatives and flavonols (B); polyamines (C); ascorbic acid, aromatic amino acids, and glycoalkaloids (D). (E, F) Individual anthocyanins in red (E) and purple (F) potatoes. The data represents the means±SEM of three biological replicates. Values with the same letter are not significantly different (*P*<0.05). The key for all panels is shown in (A). Abbreviations are listed in Supplementary Tables S1 and S2.

Individual phenylpropanoids were measured by HPLC/ESI-MS/DAD (Supplementary Table S1). 5-Chlorogenic acid (5CGA) was the predominant phenylpropanoid in all genotypes, varying from 0.12 to 6.3mg g^–1^ (Fig. 4A). Caffeoyl putrescine (CP) amounts were higher in dark yellow, red, and purple genotypes, while that of feruloyl quinic acid (FQA2) was higher in red and purple genotypes (Fig. 4B). Relative amounts of hydroxycinnamic acid amides were measured, and only *bis*-dihydrocaffeoyl spermine (BDCS), and *bis*-dihydrocaffeoyl spermidine (BDCSD) were detected in all genotypes (Fig. 4C) and levels of both were least in white potatoes.

Concentrations of shikimate-derived aromatic amino acids were lowest in the light yellow genotype (Fig. 4D). Concentrations of phenylalanine (Phe), the precursor for phenylpropanoid biosynthesis, did not associate with total phenolic or 5CGA concentrations. However, the enzyme activity of PAL, which catalyses the first committed step of phenylpropanoid pathway where cinnamic acid is formed by deamination of Phe, was about 35–45-fold higher in potatoes with higher amounts of phenylpropanoids (Supplementary Fig. S2 at *JXB* online). Another major source of tuber antioxidant capacity is ascorbic acid, which varied from 1.2 to 2.0mg g^–1^ among genotypes (Fig. 4D). Antioxidant capacity was measured by FRAP and was greater in red and purple potatoes (Supplementary Fig. S2).

### Flavonols and anthocyanins

Among these genotypes, flavonols were observed only in red and purple potatoes (Fig. 4B). Purple potatoes accumulated myricetin-3-*O*-rutionside (Myr), quercetin-3-*O*-rutinoside (Rut), and kaempferol-3-*O*-rutinoside (Kmp), and the red genotype only accumulated Kmp but in greater amounts than the total flavonols in purple potatoes. Numerous anthocyanins were present in red and purple genotypes, and the more abundant were analysed (Supplementary Table S2). Pelargonidin-3-(coumaroyl)-rutinoside-5 glucoside (PlCRG) was the predominant anthocyanin in the red genotype, contributing to >90% of total anthocyanins (Fig. 4E). In purple potatoes, petunidin-3-(coumaroyl)-rutinoside-5 glucoside (PtCRG) and peonidin-3-(coumaroyl)-rutinoside-5 glucoside (PeCRG) comprised >80% of total anthocyanins (Fig. 4F).

### Phenylpropanoid structural gene expression

**Fig. 5.**
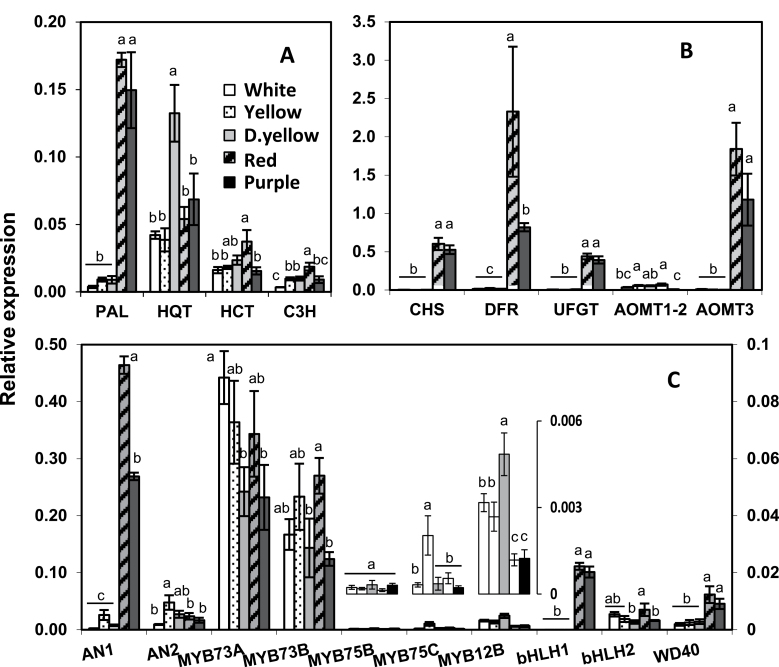
Gene expression in the five genotypes. Expression of structural genes from the early (A) and late (B) phenylpropanoid pathway, or genes encoding transcription factors (C). In (C), only *AN1* uses the left *y*-axis, and all others use the right axis. In the inset are expression values of *MYB75B*, *MYB75C*, and *MYB7512B*. The data represent the means±SEM of three biological replicates. Values with the same letter are not significantly different (*P*<0.05).

Relative expression of *PAL* was about 40-fold higher in red and purple potatoes compared with white (Fig. 5A), which is consistent with the amount of phenylpropanoids and PAL enzyme activity in these genotypes (Fig. 4 and Supplementary Fig. S2). The primary pathway to CGA biosynthesis is thought to be through hydroxycinnamoyl-CoA quinate transferase (HQT). Although 5CGA was the most abundant phenylpropanoid, *HQT* transcript expression did not track with the 5CGA levels (Fig. 5A). Expression of ρ-coumarate 3-hydroxylase (*C3H*) and hydroxycinnamoyl transferase (*HCT*) expression was 2–5-fold higher in red than in other genotypes. The expression of late genes like chalcone synthase (*CHS*), dihydroflavonol reductase (*DFR*), and UDP-glucose:flavonoid 3-*O*-glucosyltransferase (*UFGT*) were strongly expressed in red and purple genotypes and correlated with anthocyanin levels (Fig. 5B). To our knowledge, no anthocyanin *O*-methyltransferase (AOMT) gene has been reported in potato or any Solanaceae. By similarity search with grape *AOMT* (Hugueney *et al.*, 2009), three isoforms were identified and aligned (Supplementary Fig. S1D). *AOMT1* and *AOMT2* shared high similarity (90%), so only one set of primers was used for both. The expression of *AOMT1/2* showed no specific trend, while that of *AOMT3* was strongly expressed in red and purple potatoes (Fig. 5B).

### Transcription factor analysis

Expression of the 15 potato transcription factors listed in the phylogenetic analysis was studied by qRT-PCR in the flesh of the five genotypes. Expression of *MTF1*, *MTF2*, *MYB10*, *MYB12A*, and *MYB75A* were below quantifiable levels. Primers amplified an appropriately sized fragment from genomic DNA eliminating sequence compatibility as an issue. The expression data for the remaining ten transcription factors is shown in Fig. 5C. *AN1* was by far the most abundantly expressed of those analysed, and was more than 100-fold higher in the flesh of red and purple genotypes. To evaluate whether differences in the AN1 protein might account for the differential phenylpropanoid profiles observed among the five genotypes, full-length coding regions were cloned and sequenced. The sequence was identical among Challenger, AmaRosa, and Purple Majesty, whereas a white and yellow cultivar each had two nucleotide changes resulting in leucine-to-phenylalanine and leucine-to-valine substitutions (Supplementary Fig. S3a at *JXB* online).

Sequencing of ~1500bp of the *StAN1* promoter region revealed substantial differences. The promoter region was 97% similar between the low-phenylpropanoid white and yellow genotypes, and 93% similar between the purple and red potatoes. However, only 37% similarity occurred between the white/yellow and red/purple genotypes. Interestingly, the white/yellow genotypes had only one sucrose-responsive (SURE) element, whereas the red and purple potatoes had six (Supplementary Fig. S3b). Similarly, no methyl jasmonate-responsive elements were present in the promoter region examined in the white and yellow potatoes, but five were present in red and four in purple potatoes. Despite using various primers, the promoter sequence of Challenger could not be amplified.

The expression of *AN2*, *MYB75B*, and *MYB75C* was much lower and did not associate with total phenylpropanoid concentrations. Excluding *AN1*, the expression levels of *MYB73A* and *MYB73B* were higher than other transcription factors but did not associate with phenylpropanoid concentrations. *MYB12B* was poorly expressed in all genotypes, but was the only gene that showed a clear inverse association with phenylpropanoid amounts. *MYB12B* expression was 2–4-fold higher in white and yellow genotypes compared with that in red and purple potatoes.

The expression of *bHLH1* in tuber flesh was ~10–20-fold lower than that of *AN1* and was detected only in red and purple potatoes (Fig. 5C), whereas *bHLH2* was expressed in all potatoes but did not show any association with phenylpropanoid concentrations. *WD40* expression was 3–5-fold higher in the red and purple potatoes.

### Environmental effects on transcription factor expression

These results suggested that the differences in the phenylpropanoid concentrations among different cultivars were probably partly determined by *AN1*, *bHLH1*, and *WD40*, i.e. they have a role in determining the inter-genotypical variation. However, phenylpropanoid concentrations can also vary significantly within potatoes of a single genotype. Potatoes from the same cultivar grown under the same management regime can have different concentrations in different years or locations. To determine whether *AN1*, *bHLH1*, and *WD40* were also associated with intra-genotypical variation, purple potatoes grown in four locations in Alaska, plus in Texas and Florida, were examined. The Alaskan potatoes had significantly higher amounts of phenylpropanoids (Payyavula *et al.*, 2012) that correlated with higher expression of *AN1*, *bHLH1*, and *WD40* (Supplementary Fig. S4 at *JXB* online). *AN1* expression was strongest in tuber samples from Wiseman, the northern-most site located in the Arctic Circle, and least in the southern-most locations. Similar patterns were observed for *bHLH1* and *WD40*.

### Evaluation of the role of sugars in phenylpropanoid metabolism

**Fig. 6.**
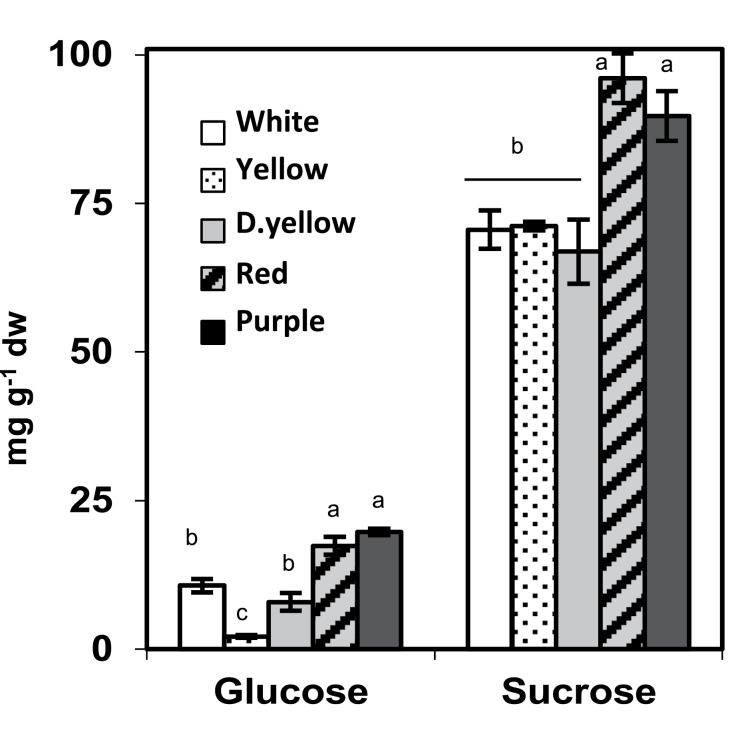
Glucose and sucrose concentrations in the five genotypes. Data represents the means±SEM of three biological replicates. Values with same letter are not significantly different (*P*<0.05).

Sugars were measured in the five genotypes (Fig. 6). The red and purple genotypes accumulated up to 30 and 60% higher sucrose and glucose levels, respectively, which is consistent with a role for sugars in modulating tuber phenylpropanoid content (Zucker and Levy, 1959; Leggewie *et al.*, 2003). On the other hand, there were differences among phenylpropanoids in white and yellow potatoes (Fig. 4A), which did not correlate with sugar content. For example, the dark yellow genotype had higher phenylpropanoid concentrations than the white genotype but equivalent amounts of sucrose and lower glucose.

**Fig. 7.**
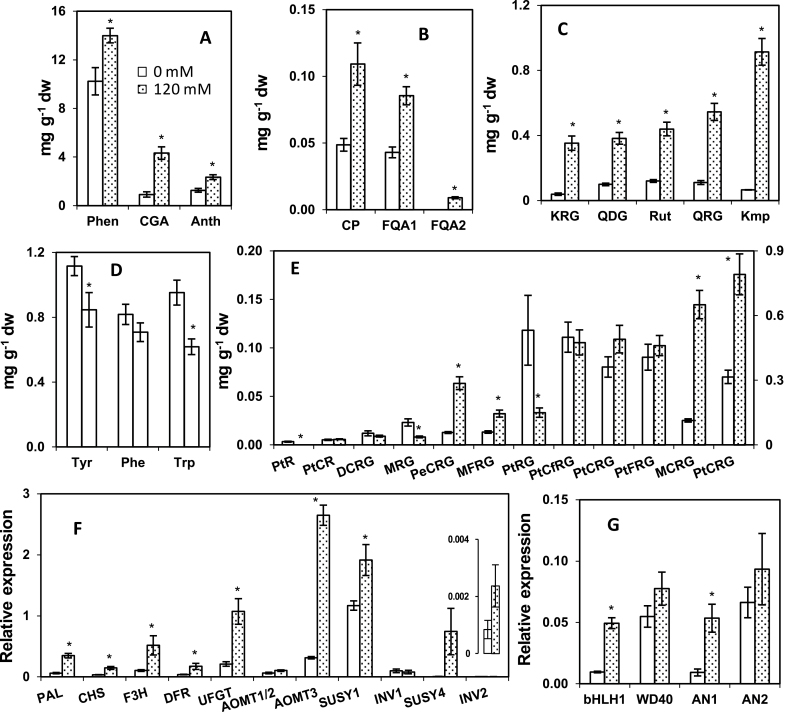
Metabolite and gene expression changes associated with sucrose feeding. (A–E) Amounts of total phenolics, total chlorogenic acids (5CGA plus isomers), and total anthocyanins (A), caffeoyl and feruloyl derivatives (B), individual flavonols (C), aromatic amino acids (D), and anthocyanins (E). PtCRG is shown on the right *y*-axis, and all others on the left. (F, G) Expression of phenylpropanoid and sugar-related genes, with expression of *INV2* shown in the inset (F), and transcription factors in potato plantlets (G) cultured on 0mM (open bars) and 120mM (dotted bars) sucrose. Data represents the means±SEM of three biological replicates. Asterisks indicate treatments that are significantly different (*P*<0.05).

Preliminary sugar feeding studies showed increases in phenylpropanoids in both white and purple potatoes. Subsequently, the cultivar Purple Majesty was selected for in-depth analysis, and the effect of sucrose on metabolites and gene expression was studied in plantlets cultured on 0 and 120mM sucrose (Fig. 7). Total phenolics increased by 40% reaching 14mg g^–1^ in 120mM sucrose-treated plantlets (Fig. 7A). Sucrose induced an almost 5-fold increase in total chlorogenic acid content. Numerous other phenylpropanoids including multiple flavonols showed a strong induction by sucrose, with the most abundant flavonol, Kmp, induced almost 15-fold (Fig. 7A–C). Likewise, the anthocyanin branch of the pathway was strongly induced, with total anthocyanins increasing by ~85% (Fig. 7A). PtCRG was the predominant anthocyanin and increased by 2.5-fold with sucrose feeding (Fig. 7E). Among the less-abundant anthocyanins, some decreased in response to sucrose, such as petunidin 3-rutinoside-5-glucoside (PtRG), which may reflect competition with the more abundant anthocyanins for common precursors. Unlike a majority of the phenylpropanoids examined, the three shikimate-derived aromatic amino acids decreased in sucrose-fed plantlets (Fig. 7D).

Expression of the structural genes *PAL*, *CHS*, *F3H*, *DFR*, *UFGT*, *AOMT3*, and transcription factors *AN1* and *bHLH1* was more than 3-fold higher in plantlets fed 120mM sucrose, but *WD40* and *AN2* showed only a slight increase (Fig. 7F, G). Genes encoding isoforms of the sucrose-hydrolysing enzymes sucrose synthase (SUSY) and invertase (INV) were also studied. The expression of *SUSY1* was several fold higher than *SUSY4.* Both were induced by sucrose, and *SUSY4* underwent a >100-fold increase in expression after sucrose feeding (Fig. 7F). Expression of *INV1* was greater than *INV2*, but did not increase in response to sucrose, whereas *INV2* expression increased.

### Transient leaf infiltration assays

**Fig. 8.**
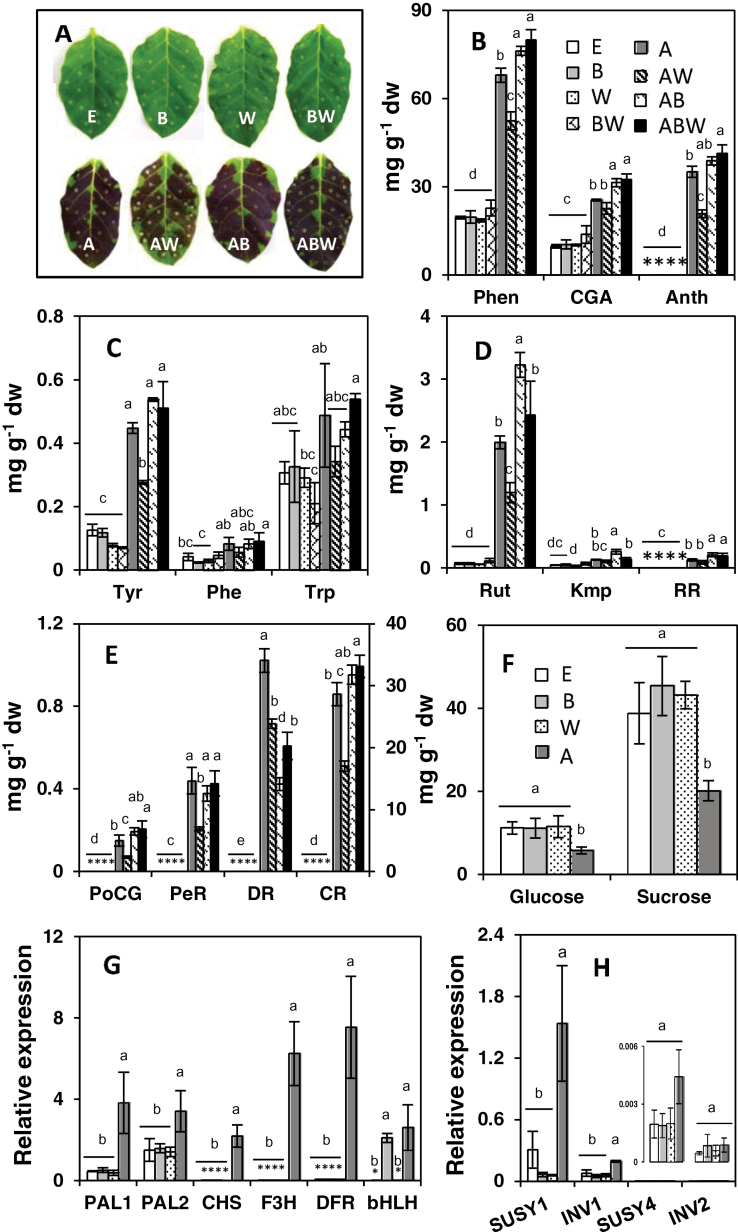
Metabolite and gene expression in transient leaf infiltration assays. (A) Tobacco leaves infiltrated with individual constructs or combinations of the four constructs. The letter on each leaf indicates the construct(s) infiltrated into that leaf. E, empty vector; B, *bHLH1*; W, *WD40*; A, *AN1*. (B) Levels of total phenolics, chlorogenic acids, and anthocyanins in leaves infiltrated with the designated construct(s). (C–F) Levels of aromatic amino acids (C), flavonols (D), and individual anthocyanins (E), and sugars (F). CR in (E) uses the *y*-axis on the right. The key in (B) also applies to (C)–(E). (G) Expression of phenylpropanoid genes and *bHLH1. PAL1* is *PAL1*+*PAL4* and *PAL2* is *PAL2*+*PAL3.* (H) Sucrolytic gene expression in tobacco leaves infiltrated with different constructs. Expression of *SUSY4* and *INV2* is shown in the inset. The key for (G) and (H) is shown in (F). Asterisks indicate compounds below the limit of detection. The data represent the means±SEM of three biological replicates. Values with same letter are not significantly different (*P*<0.05).

The functionality of the selected potato transcription factors was tested using tobacco leaf infiltration. Leaves infiltrated with empty vector, *bHLH*, *WD40*, or combined *bHLH* plus *WD40* did not show pigmentation (Fig. 8A). Anthocyanin accumulation was time dependent and all leaves infiltrated with any combination of *AN1* showed pigmentation by 96h post-infiltration (Fig. 8A). However, at 48h, visible purple pigmentation was only observed on leaves co-infiltrated with *AN1+bHLH* (Supplementary Fig. S5 at *JXB* online). This accelerated anthocyanin induction was observed across multiple independent experiments. After 72h, leaves infiltrated with *AN1* alone or *AN1+WD40* constructs also accumulated purple pigmentation, but were less intense than leaves infiltrated with *AN1+bHLH* or *AN1+bHLH1+WD40*.

Phenylpropanoid profiles in leaves infiltrated with *bHLH1*, *WD40*, or *bHLH1+WD40* were similar to those infiltrated with empty vector, but *AN1* or any of its combinations induced 2–4-fold higher levels of total phenolics and CGA (Fig. 8B). Interestingly, concentrations of the three aromatic amino acids increased with *AN1* infiltration or its combinations (Fig. 8C). Rhamnetin rutinoside (RR) accumulated with *AN1* infiltration, and rutin, the major flavonol, increased ~30-fold (Fig. 8D).

Cyanidin 3-rutinoside (CR) was by far the most abundant anthocyanin induced in *N. tabacum* (Fig. 8E and Supplementary Fig. S6A, E at *JXB* online). In contrast, when *N. benthamiana* leaves were infiltrated with *StAN1*, the major anthocyanin was delphinidin 3-rutinoside (DR; Supplementary Fig. S6B, F). Purple and blue potatoes contain petunidin and malvinidin derivatives, which are methylated products of delphinidin (Fig. 4F; Hillebrand *et al.*, 2009). A possible reason for not forming downstream products of delphinidin in tobacco could be due to a lack of AOMT activity. To address this question and address the functionality of the potato gene, *N. benthamiana* leaves were infiltrated with potato *AOMT3* alone or in combination with *AN1*. Anthocyanins were not observed in leaves infiltrated with *AOMT3* alone (Supplementary Fig. S6C), but when infiltrated with *AOMT3* and *AN1*, the amounts of DR were greatly reduced, and two major peaks appeared of *m*/*z* 625 and 639 (M+H)^+^ that were identified as the methylated anthocyanins petunidin 3-rutinoside (PtR) and malvidin 3-rutinoside (MR) (Supplementary Fig. S6D, G, H).

From the above data, it was clear that the metabolite changes were predominantly due to *AN1* infiltration, so subsequent experiments did not use combinatorial infiltrations. Changes in sucrose and glucose levels, PAL activity, and the expression of several key phenylpropanoid genes, transcription factors, and sugar metabolism genes were estimated in leaves infiltrated with empty vector, *bHLH1*, *WD40*, or *AN1* (Fig. 8F–H and Supplementary Fig. S7 at *JXB* online). In tobacco, four *PAL* isoforms have been reported, of which *PAL1* and *PAL4*, and *PAL2* and *PAL3* have high similarity (Reichert *et al.*, 2009). Therefore, *PAL1* and *PAL4* were amplified together with only one primer set, as were *PAL2* and *PAL3*. Expression of both *PAL1/4* and *PAL2/3* increased, along with the late pathway genes *CHS*, *F3H*, and *DFR* in *AN1*-infiltrated samples (Fig. 8G). PAL enzyme activity increased more than 25-fold in *AN1*-infiltrated samples (Supplementary Fig. S7). Notably, the expression of *NtbHLH* was increased in *AN1-*infiltrated samples. In leaves infiltrated with *StbHLH1*, the expression of *bHLH1* increased, probably because *NtbHLH* primers amplified *StbHLH1*, which has a high sequence similarity.

Strikingly, infiltration with *AN1* induced a marked change in sugar metabolism, as seen by the stimulation of *SUSY1*, *SUSY4*, and *INV1* expression (Fig. 8H) and the sizeable decrease in sucrose and glucose concentrations (Fig. 8F).

### Promoter elements

The regulatory elements in the promoters of selected potato genes were predicted in the 1500bp sequence upstream of ATG using the PLACE database (Higo *et al.*, 1999). We were not able to retrieve promoter sequences for *UFGT* and *AOMT3*. The interaction of transcription factors and sugars was of particular interest; therefore, MYB, bHLH, and SURE elements were examined. The occurrence of the three MYB recognition sites, MYBCORE (CNGTTR; Solanol *et al.*, 1995), MYBPLANT (MACCWAMC; Sablowski *et al.*, 1994), and MYBPZM (CCWACC; Grotewold *et al.*, 1994); one bHLH recognition site (G-box) MYCCONSENSUS (CANNTG; Blackwell and Weintraub, 1990; Hartmann *et al.*, 2005); and one SURE (AATAGAAAA; Grierson *et al.*, 1994) for different gene promoters is shown in Supplementary Table S5 at *JXB* online. MYBCORE was present in all the promoters except *INV1* and was over-represented in *WD40* and *SUSY1*. MYCCONSENSUS was the most abundantly represented element and was present multiple times (two to nine) in all the promoters except *HQT*. SURE was present only in A*N1*, *WD40*, *SUSY1*, *HCT*, and *HQT* (Supplementary Table S5).

## Discussion

Relatively few studies have focused on the regulation of phenylpropanoid metabolism in a tuber crop. To identify candidate transcription factors that regulate the phenylpropanoid pathway, metabolite and gene expression profiles were determined in tubers of five genotypes with decidedly different phenylpropanoid profiles (Fig. 3B). Field-grown tubers were used so results would relate to the crop and avoid the potentially non-representative results noted previously in studies using greenhouse-grown potatoes (Chawla *et al.*, 2012; Navarre *et al.*, 2013). Peeled samples were used to more clearly differentiate the samples by ensuring that all samples represented distinct flesh colours and not a mix such as red skin and white flesh. Moreover, phenylpropanoid metabolism differs between tuber skin and flesh, and less is known about the flesh (Jung *et al.*, 2005) than periderm, in which genetic analysis has revealed three major loci controlling pigmentation, *D*, *R*, and *P* (De Jong *et al.*, 2004).

### AN1, bHLH1, *and* WD40 *regulate tuber anthocyanins and other phenylpropanoids*

Red and purple potatoes had higher expression of phenylpropanoid structural genes and contained higher amounts of phenylpropanoids, not just anthocyanins but colourless compounds such as 5CGA (Figs 3B and 4A, B). Compared with concentrations reported previously in transgenic tubers overexpressing the R2R3 MYB *StMTF1* (Rommens *et al.*, 2008), the wild-type red and purple potatoes in this study contained higher levels of phenylpropanoids, including almost 4-fold and 2-fold higher amounts of 5CGA and Kmp, respectively. Analysis of the potential involvement of 15 transcription factors was measured by qRT-PCR. *AN1* was the most abundantly expressed transcription factor and was >100-fold higher in red and purple potatoes than in white. Besides *AN1*, only two of the other examined transcription factors, *bHLH1* and *WD40*, showed a positive correlation with phenylpropanoid concentrations (Supplementary Fig. S8 at *JXB* online). Over 30 phenylpropanoid-related compounds were measured, of which most were only minor components of total soluble tuber phenylpropanoids in red and purple potatoes (Fig. 4). Notably 5CGA and a single anthocyanin accounted for 80–90% of the total. Amounts of CGA and total phenylpropanoids strongly correlated with *AN1* expression in genotypes with different phenylpropanoid profiles, and in response to environmental signals, sucrose feeding, and *AN1* infiltration. These results suggested that AN1 can mediate marked changes in phenylpropanoids outside the anthocyanin pathway, as chlorogenic acid is synthesized by a different branch of the pathway from anthocyanins. Consistent with this finding is the fact that 5CGA is found in much higher amounts in red and purple potatoes than in white or yellow (André *et al.*, 2007b; Navarre *et al.*, 2011).

To our knowledge, this is the first report of a potato *WD40* whose expression correlates with phenolics and anthocyanins (Supplementary Fig. S8). *WD40* was not able to induce phenylpropanoid expression on its own in infiltration studies (Fig. 8A), consistent with previous results where overexpression of WD40 from *Medicago truncatula* (*MtWD40*) in hairy roots failed to induce flavonols, proanthocyanidins, or anthocyanins (Pang *et al.*, 2009). In contrast, overexpression of *MtWD40* was able to complement the lack of red-pigmented phenotype of a mutant line NF0977, which suggests that WD40 is required but not sufficient for anthocyanin pathway activation (Pang *et al.*, 2009). However, ectopic expression of a grape WD40 induced anthocyanins in *Arabidopsis* leaves (Matus *et al.*, 2010). These results illustrate the complex interactions among the transcription factors.

*bHLH1* but not *bHLH2* showed a strong association with phenylpropanoid expression. A quantitative trait locus study linked a potato *bHLH* to anthocyanin synthesis and reported that it was expressed in all the coloured genotypes and in 21 of 53 white or yellow genotypes, suggesting that it is required but not sufficient for anthocyanin synthesis (Zhang *et al.*, 2009b). While we were unable to determine if these *bHLH*s are the same because sequence information was not available for the previous *bHLH*, both are localized on chromosome 9. The expression patterns of *AN1*, *bHLH1*, and *WD40* suggest that they are determinants of the amounts of phenylpropanoids that a given genotype will contain. *AN1*, *bHLH1*, and *WD40* were also implicated in the intra-genotypical variation that occurs in potatoes in response to environmental variations, such as when the same purple genotype was grown in Alaska, Texas, and Florida (Supplementary Fig. S4). The higher expression in the Alaskan-grown potatoes might be due to the lower temperatures or longer day length, and potentially reflects greater stress.

Unlike a previous microarray study that reported an ~40-fold increase in *MYB73* in the purple flesh of a sectored potato that had both white and purple regions (Stushnoff *et al.*, 2010), elevated expression of *MYB73* was not observed in the red or purple genotypes relative to the white or yellow potatoes (Fig. 5C). *MYB12*, which regulates the flavonol branch of the pathway in *Arabidopsis* (Mehrtens *et al.*, 2005), appeared to be negatively associated with tuber phenylpropanoid content. The minimal repression domain (TLLLFR) present at the C terminus of AtMYBL2, a negative regulator of anthocyanin biosynthesis in *Arabidopsis* (Matsui *et al.*, 2008), was not found in StMYB12B or in other MYB transcription factors in this study. Nor was the ERF-associated amphiphilic repression (EAR) motif, associated with repression of anthocyanin gene transcription, present in any in these potato MYBs (Ohta *et al.*, 2001; Lin-Wang *et al.*, 2011). Expression of other transcription factors varied among the genotypes but did not seem to be associated with phenylpropanoid content. Among the potato MYBs examined, StMTF1 has the highest percentage similarity with AtPAP1 (Supplementary Table S4); however, the *StMTF1* transcript was not present at detectable levels in any of the genotypes examined, suggesting that the native gene does not have a major role in tuber phenylpropanoid metabolism. A previous study showing a stimulatory effect of *StMTF1* (Rommens *et al.*, 2008) may be due to its being expressed under a heterologous promoter. Potentially *StMTF1* under its own promoter is active in other tissues or conditions.

Importantly, the stimulatory effects of *AN1* on phenylpropanoid metabolism were not limited to anthocyanins because other compounds were also upregulated, notably 5CGA, the predominant soluble phenylpropanoid in potatoes (Fig. 8B). The increase in expression of *CHS* and *F3H* (Fig. 8G) along with a 30-fold increase in Rut (Fig. 8D) suggested that *StAN1* upregulates 3′-hydroxylated flavonones. Collectively, the relationship between *StAN1* expression and high pelargonidin and petunidin concentrations in coloured potatoes and the induction of cyanidin or delphinidin derivatives in *StAN1*-infiltrated *N. tabacum* or *N. benthamiana* leaves suggest that StAN1 regulates flavanone 3-hydroxylase (F3H), flavonoid 3-hydroxylase (F3′H), and flavonoid 3′,5′-hydroxylase (F3′5′H).

Differences were seen in aromatic amino acid pools among the samples. No obvious relationship was observed between these amino acids and phenylpropanoids in the field-grown tubers, whereas sucrose feeding lowered aromatic amino acid concentrations but increased phenylpropanoids, *AN1*, *bHLH1*, and *WD40* expression. However, *AN1* infiltration decreased sucrose and glucose but increased the amounts of aromatic amino acids. The increase was particularly clear with tyrosine and not quite so dramatic with phenylalanine and tryptophan. Thus, sucrose feeding and *AN1* infiltration both increased phenylpropanoids, whereas sucrose treatments decreased aromatic amino acid amounts but *AN1* increased them. Understanding the basis for these differences will require further study.

### AOMT

To our knowledge, a gene for AOMT has not been reported previously in potatoes or other solanaceous plants. *AOMT3* but not *AOMT1/2* showed a strong correlation with anthocyanins (Supplementary Fig. S8) and altered anthocyanin profiles when infiltrated into leaves. AOMT3 probably plays a key role in determining the type of anthocyanins that accumulate in potatoes, and its identification offers another potential target for efforts to manipulate tuber anthocyanin composition. The failure of *N. benthamiana* leaves to synthesize methylated anthocyanins when infiltrated with *StAN1* suggests that either AN1 does not regulate AOMT or that tobacco leaves lack a functional AOMT. BLAST searches for an *AOMT3* homologue in tobacco did not identify any candidates.

### Sucrose induces AN1 and phenylpropanoid biosynthesis

In addition to providing carbon for phenylpropanoid metabolism, sugars also regulate anthocyanin biosynthesis (Teng *et al.*, 2005). During grape berry development, the increase in sugars modulates expression of anthocyanin biosynthetic genes (Boss *et al.*, 1996). High-phenylpropanoid red and purple potatoes had substantially higher amounts of sucrose and glucose than white or yellow tubers (Fig. 6). Similar relationships were seen in a tuber developmental study in which sugar and phenylpropanoid concentrations were correlated (Navarre *et al.*, 2013). To gain direct evidence, potato plantlets were cultured on medium supplemented with 0 or 120mM sucrose, resulting in large increases in phenylpropanoids (Fig. 7A). Sucrose stimulated significant increases in the expression of *AN1*, *bHLH1*, and *WD40*, demonstrating that the stimulatory effect of sucrose on potato phenylpropanoid metabolism is at least partly modulated through these transcription factors. Supporting this finding is the higher expression of *AN1*, *bHLH1*, and *WD40* in the field-grown potatoes that had highest sucrose and phenylpropanoid concentrations.

The presence of SURE elements in the promoter of *AN1* (Supplementary Fig. S3b) is consistent with its regulation by sucrose. In addition to greater amounts of sucrose, purple and red potatoes contained six SURE elements in the AN1 promoter, whereas white and yellow potatoes had a single SURE element. A mechanism for the 5-fold increase in CGA (Fig. 7A) in response to sucrose is suggested by the *MYB*, *bHLH*, and *SURE* elements present in the promoter of *HQT*. SURE elements were not observed in the *bHLH1* promoter, but *bHLH1* expression was upregulated by sucrose treatment. A possible explanation is that the potato *AN1* induced expression of tobacco *bHLH* in transient assays (Fig. 8G), which is consistent with a previous report of tobacco transcription factors (Bai *et al.*, 2011). *AN1*-infiltrated leaves showed a 24h delay in anthocyanin formation compared with *AN1+bHLH1* co-infiltrated leaves (Supplementary Fig. S5), and the delay may reflect the time needed for *AN1* to recruit *bHLH*. The promoters of potato *PAL*, *CHS*, *F3H*, and *DFR* did not have SURE elements, but had MYB and bHLH regulatory elements (Supplementary Table S5), suggesting that the increased expression after sucrose feeding was due to AN1 and bHLH1. Anthocyanin levels in potato correlated with the expression of *ANS* and *UFGT* (Hu *et al.*, 2011; Keifenheim *et al.*, 2006) and higher expression of these genes was observed in this study in the high-phenylpropanoid potatoes and sucrose-treated potato plantlets (Figs 5B and 7F).

**Fig. 9.**
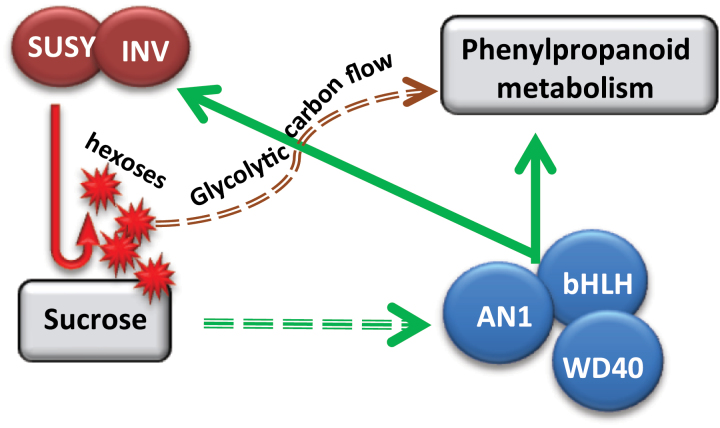
Proposed model for the interaction of AN1, sucrose, and sucrose metabolic genes to modulate phenylpropanoid metabolism. Sucrose stimulates *AN1* expression, which with *bHLH1* and *WD40* regulates phenylpropanoid expression, but also sucrolytic genes that can provide carbon for induced phenylpropanoid metabolism and decrease sucrose. The red line indicates the cleavage of sucrose. Solid green lines indicate targets stimulated by AN1. The dashed green arrow shows *AN1* expression stimulated by sucrose concentrations, which can decrease in response to *AN1* expression.

Interestingly, sucrose treatments of potato plantlets induced large increases in *AN1* and *bHLH1* expression, and to a lesser extent that of *WD40* (Fig. 7G), whereas infiltration of potato *AN1* into tobacco leaves increased expression of *SUSY* and *INV* genes (Fig. 8H). This suggests the possibility of a regulatory loop in which sucrose increases *AN1* expression but *AN1* decreases sucrose concentrations by inducing sucrolytic enzymes that liberate hexoses that are channelled to the phenylpropanoid pathway (Fig. 9). In contrast, *bHLH1* and *WD40* infiltration alone did not increase expression of SUSY or INV or mobilize sucrose. The extent that sucrose–AN1 interactions modulate tuber phenylpropanoid metabolism is an interesting question that awaits future research.

In addition to their important *in planta* roles, phenylpropanoids are desirable in the diet because of their health-promoting properties, which include antioxidant, anti-inflammatory, hypotensive, and chemopreventative effects (Manach *et al.*, 2004; Prior, 2003; Kaspar *et al.*, 2011; Vinson *et al.*, 2012). High-phenylpropanoid potatoes would be especially valuable because of the high consumption of this staple food. The potential of potatoes to provide dietary phenylpropanoids, including anthocyanins, is significant. For example, the purple potatoes in this study contained anthocyanin amounts that approach those of high-anthocyanin transgenic tomatoes (Butelli *et al.*, 2008), and exceed amounts in many fruits and vegetables (see Table 2 in Wu *et al.*, 2006). Collectively, these data suggest that interactions among sucrose, sucrolytic enzymes, and AN1 modulate the pathway, and that AN1 is a key regulator of the most abundant tuber phenylpropanoids. Increased understanding of tuber phenylpropanoid metabolism will facilitate efforts to develop potatoes with optimal types and concentrations of phenylpropanoids.

## Supplementary data

Supplementary data are available at JXB online.

Supplementary Fig. S1. Protein sequence alignment of representative (A) MYBs, (B) bHLH, (C) WD40, and (D) AOMT from potato and from other species.

Supplementary Fig. S2. PAL enzyme activity and FRAP antioxidant activity in the five genotypes.

Supplementary Fig. S3. (A) StAN1 protein sequence of the five potato genotypes used in this study. (B) *StAN1* promoter region from four genotypes used in this study.

Supplementary Fig. S4. Expression of selected transcription factors in purple potatoes grown in Alaska (Wiseman, Fairbanks, Palmer and Juneau), Texas and Florida.

Supplementary Fig. S5. Tobacco leaves 48 and 72h after infiltrating with a binary construct harbouring *AN1* (A) alone or *AN1* + *bHLH1* (AB).

Supplementary Fig. S6. MS extracted ion data of the most abundant anthocyanins formed in (A) *N. tabacum* (Samsun) infiltrated with *AN1*. (B) *N. benthamiana* leaves infiltrated with *AN1*, (C) *AOMT3*, and (D) *AN1*+*AOMT3*.

Supplementary Fig. S7. PAL activity in tobacco leaves infiltrated with (E) empty vector, (B) *bHLH1*, (W) *WD40*, or (A) *AN1*.

Supplementary Fig. S8. Correlation analysis of transcript and metabolite expression in tubers from five potato genotypes.

Supplementary Table S1. Retention times and MS data of select compounds present in potato phenolic extracts separated by HPLC.

Supplementary Table S2. Retention times and MS data of anthocyanins in potato extracts separated by HPLC.

Supplementary Table S3. List of primers used in this study.

Supplementary Table S4. Protein similarity matrix of transcription factors from different species.

Supplementary Table S5. List of regulatory elements in promoters of different genes from *S. tuberosum* group Phureja.

Supplementary Data

## Abbreviations:

bHLH: basic helix–loop–helix
ESI: electrospray ionization
FRAP: ferric reducing antioxidant power
LC: liquid chromatography
MS: mass spectrometry
PGSC: Potato Genome Sequencing Consortium
qRT-PCR: quantitative real-time PCR.

## References

[CIT0001] André CM Ghislain M Bertin P Oufir M Herrera Mdel R Hoffmann L Hausman JF Larondelle Y Evers D 2007a Andean potato cultivars (*Solanum tuberosum* L.) as a source of antioxidant and mineral micronutrients. Journal of Agricultural and Food Chemistry 55, 366–378 17227067 10.1021/jf062740i

[CIT0002] André CM Oufir M Guignard C Hoffmann L Hausman JF Evers D Larondelle Y 2007b Antioxidant profiling of native Andean potato tubers (*Solanum tuberosum* L.) reveals cultivars with high levels of beta-carotene, alpha-tocopherol, chlorogenic acid, and petanin. Journal of Agricultural and Food Chemistry 55, 10839–10849 18044831 10.1021/jf0726583

[CIT0003] André CM Schafleitner R Legay S Lefèvre I Aliaga CAA Nomberto G Hoffmann L Hausman J-F Larondelle Y Evers D 2009 Gene expression changes related to the production of phenolic compounds in potato tubers grown under drought stress. Phytochemistry 70, 1107–1116 19664789 10.1016/j.phytochem.2009.07.008

[CIT0004] Bai Y Pattanaik S Patra B Werkman J Xie C Yuan L 2011 Flavonoid-related basic helix-loop-helix regulators, NtAn1a and NtAn1b, of tobacco have originated from two ancestors and are functionally active. Planta 234, 363–375 21484270 10.1007/s00425-011-1407-y

[CIT0005] Ballester AR Molthoff J de Vos R 2010 Biochemical and molecular analysis of pink tomatoes: deregulated expression of the gene encoding transcription factor SlMYB12 leads to pink tomato fruit color. Plant Physiology 152, 71–84 19906891 10.1104/pp.109.147322 PMC2799347

[CIT0006] Benzie IFF Strain JJ 1996 The ferric reducing ability of plasma (FRAP) as a measure of “antioxidant power”: the FRAP assay. Analytical Biochemistry 239, 70–76 8660627 10.1006/abio.1996.0292

[CIT0007] Blackwell T Weintraub H 1990 Differences and similarities in DNA-binding preferences of MyoD and E2A protein complexes revealed by binding site selection. Science 250, 1104–1110 2174572 10.1126/science.2174572

[CIT0008] Borovsky Y Oren-Shamir M Ovadia R Jong W Paran I 2004 The *A* locus that controls anthocyanin accumulation in pepper encodes a *MYB* transcription factor homologous to *Anthocyanin2* of Petunia. Theoretical and Applied Genetics 109, 23–29 14997303 10.1007/s00122-004-1625-9

[CIT0009] Boss PK Davies C Robinson SP 1996 Analysis of the expression of anthocyanin pathway genes in developing *Vitis vinifera* L. cv Shiraz grape berries and the implications for pathway regulation. Plant Physiology 111, 1059–1066 12226348 10.1104/pp.111.4.1059 PMC160981

[CIT0010] Butelli E Titta L Giorgio M 2008 Enrichment of tomato fruit with health-promoting anthocyanins by expression of select transcription factors. Nature Biotechnology 26, 1301–1308 10.1038/nbt.1506 18953354

[CIT0011] Chang SJ Puryear J Cairney J 1993 A simple and efficient method for isolating RNA from pine trees. Plant Molecular Biology Reporter 11, 113–116

[CIT0012] Chawla R Shakya R Rommens CM 2012 Tuber-specific silencing of asparagine synthetase-1 reduces the acrylamide-forming potential of potatoes grown in the field without affecting tuber shape and yield. Plant Biotechnology Journal 10, 913–924 22726556 10.1111/j.1467-7652.2012.00720.x

[CIT0013] Chun OK Kim DO Smith N Schroeder D Han JT Lee CY 2005 Daily consumption of phenolics and total antioxidant capacity from fruit and vegetables in the American diet. Journal of the Science of Food and Agriculture 85, 1715–1724

[CIT0014] Coutu C Brandle J Brown D Brown K Miki B Simmonds J Hegedus D 2007 pORE: a modular binary vector series suited for both monocot and dicot plant transformation. Transgenic Research 16, 771–781 17273915 10.1007/s11248-007-9066-2

[CIT0015] De Jong WS Eannetta NT De Jong DM Bodis M 2004 Candidate gene analysis of anthocyanin pigmentation loci in the Solanaceae. Theoretical and Applied Genetics 108, 423–432 14523517 10.1007/s00122-003-1455-1

[CIT0016] Dubos C Stracke R Grotewold E Weisshaar B Martin C Lepiniec L 2010 MYB transcription factors in Arabidopsis. Trends in Plant Science 15, 573–581 20674465 10.1016/j.tplants.2010.06.005

[CIT0017] Espley RV Hellens RP Putterill J Stevenson DE Kutty-Amma S Allan AC 2007 Red colouration in apple fruit is due to the activity of the MYB transcription factor, MdMYB10. The Plant Journal 49, 414–427 17181777 10.1111/j.1365-313X.2006.02964.x PMC1865000

[CIT0018] Feyissa D Lovdal T Olsen K Slimestad R Lillo C 2009 The endogenous GL3, but not EGL3, gene is necessary for anthocyanin accumulation as induced by nitrogen depletion in *Arabidopsis* rosette stage leaves. Planta 230, 747–754 19621239 10.1007/s00425-009-0978-3

[CIT0019] Gollop R Even S Colova-Tsolova V Perl A 2002 Expression of the grape dihydroflavonol reductase gene and analysis of its promoter region. Journal of Experimental Botany 53, 1397–1409 12021287

[CIT0020] Grant GA 2006 The ACT domain: A small molecule binding domain and its role as a common regulatory element. Journal of Biological Chemistry 281, 33825–33829 16987805 10.1074/jbc.R600024200

[CIT0021] Grierson C Du J-S De Torres Zabala M Beggs K Smith C Holdsworth M Bevan M 1994 Separate *cis* sequences and *trans* factors direct metabolic and developmental regulation of a potato tuber storage protein gene. The Plant Journal 5, 815–826 8054988 10.1046/j.1365-313x.1994.5060815.x

[CIT0022] Grotewold E Drummond BJ Bowen B Peterson T 1994 The myb-homologous P gene controls phlobaphene pigmentation in maize floral organs by directly activating a flavonoid biosynthetic gene subset. Cell 76, 543–553 8313474 10.1016/0092-8674(94)90117-1

[CIT0023] Grotewold E Sainz MB Tagliani L Hernandez JM Bowen B Chandler VL 2000 Identification of the residues in the Myb domain of maize C1 that specify the interaction with the bHLH cofactor R. Proceedings of the Academy of Sciences, USA 97, 13579–13584 10.1073/pnas.250379897 PMC17618 11095727

[CIT0024] Grotewold E 2005 Plant metabolic diversity: a regulatory perspective. Trends in Plant Science 10, 57–62 15708342 10.1016/j.tplants.2004.12.009

[CIT0025] Hartmann U Sagasser M Mehrtens F Stracke R Weisshaar B 2005 Differential combinatorial interactions of *cis*-acting elements recognized by R2R3-MYB, BZIP, and BHLH factors control light-responsive and tissue-specific activation of phenylpropanoid biosynthesis genes. Plant Molecular Biology 57, 155–171 15821875 10.1007/s11103-004-6910-0

[CIT0026] Higo K Ugawa Y Iwamoto M Korenaga T 1999 Plant *cis*-acting regulatory DNA elements (PLACE) database: 1999. Nucleic Acids Research 27, 297–300 9847208 10.1093/nar/27.1.297 PMC148163

[CIT0027] Hillebrand S Naumann H Kitzinski N Kohler N Winterhalter P 2009 Isolation and characterization of anthocyanins from blue-fleshed potatoes (*Solanum tuberosum* L.). Food 3, 96–101

[CIT0028] Hu C Gong Y Jin S Zhu Q 2011 Molecular analysis of a UDP-glucose: flavonoid 3-*O*-glucosyltransferase (UFGT) gene from purple potato (*Solanum tuberosum*). Molecular Biology Reports 38, 561–567 20358295 10.1007/s11033-010-0141-z

[CIT0029] Hugueney P Provenzano S Verries C Ferrandino A Meudec E Batelli G Merdinoglu D Cheynier V Schubert A Ageorges A 2009 A novel cation-dependent *O*-methyltransferase involved in anthocyanin methylation in grapevine. Plant Physiology 150, 2057–2070 19525322 10.1104/pp.109.140376 PMC2719152

[CIT0030] Jung CS Griffiths HM De Jong DM Cheng S Bodis M De Jong WS 2005 The potato *P* locus codes for flavonoid 3′,5′-hydroxylase. Theoretical and Applied Genetics 110, 269–275 15565378 10.1007/s00122-004-1829-z

[CIT0031] Jung CS Griffiths HM De Jong DM Cheng S Bodis M Kim TS De Jong WS 2009 The potato developer (*D*) locus encodes an R2R3 MYB transcription factor that regulates expression of multiple anthocyanin structural genes in tuber skin. Theoretical and Applied Genetics 120, 45–57 19779693 10.1007/s00122-009-1158-3 PMC2778721

[CIT0032] Kaspar KL Park JS Brown CR Mathison BD Navarre DA Chew BP 2011 Pigmented potato consumption alters oxidative stress and inflammatory damage in men. Journal of Nutrition 141, 108–111 21106930 10.3945/jn.110.128074

[CIT0033] Keifenheim D Smith A Tong C 2006 Cloning and accumulation of anthocyanin biosynthesis genes in developing tubers. American Journal Potato Research 83, 233–239

[CIT0034] Koch KE 1996 Carbohydrate-modulated gene expression in plants. Annual Review of Plant Physiology and Plant Molecular Biology 47, 509–540 10.1146/annurev.arplant.47.1.509 15012299

[CIT0035] Koes R Verweij W Quattrocchio F 2005 Flavonoids: a colorful model for the regulation and evolution of biochemical pathways. Trends in Plant Science 10, 236–242 15882656 10.1016/j.tplants.2005.03.002

[CIT0036] Leggewie G Kolbe A Lemoine R 2003 Overexpression of the sucrose transporter SoSUT1 in potato results in alterations in leaf carbon partitioning and in tuber metabolism but has little impact on tuber morphology. Planta 217, 158–167 12721860 10.1007/s00425-003-0975-x

[CIT0037] Lin-Wang K Micheletti D Palmer J 2011 High temperature reduces apple fruit colour via modulation of the anthocyanin regulatory complex. Plant, Cell & Environment 34, 1176–1190 10.1111/j.1365-3040.2011.02316.x 21410713

[CIT0038] Livak KJ Schmittgen TD 2001 Analysis of relative gene expression data using real-time quantitative PCR and the 2–^ΔΔ*C*T^ method. Methods 25, 402–408 11846609 10.1006/meth.2001.1262

[CIT0039] Luo J Butelli E Hill L Parr A Niggeweg R Bailey P Weisshaar B Martin C 2008 AtMYB12 regulates caffeoyl quinic acid and flavonol synthesis in tomato: expression in fruit results in very high levels of both types of polyphenol. The Plant Journal 56, 316–326 18643978 10.1111/j.1365-313X.2008.03597.x

[CIT0040] Manach C Scalbert A Morand C Remesy C Jimenez L 2004 Polyphenols: food sources and bioavailability. American Journal of Clinical Nutrition 79, 727–747 15113710 10.1093/ajcn/79.5.727

[CIT0041] Mathews H Clendennen SK Caldwell CG 2003 Activation tagging in tomato identifies a transcriptional regulator of anthocyanin biosynthesis, modification, and transport. Plant Cell 15, 1689–1703 12897245 10.1105/tpc.012963 PMC167162

[CIT0042] Matsui K Umemura Y Ohme-Takagi M 2008 AtMYBL2, a protein with a single MYB domain, acts as a negative regulator of anthocyanin biosynthesis in *Arabidopsis* . The Plant Journal 55, 954–967 18532977 10.1111/j.1365-313X.2008.03565.x

[CIT0043] Matus JT Poupin MJ Canon P Bordeu E Alcalde JA Arce-Johnson P 2010 Isolation of WDR and bHLH genes related to flavonoid synthesis in grapevine (*Vitis vinifera* L.). Plant Molecular Biology 72, 607–620 20112051 10.1007/s11103-010-9597-4

[CIT0044] Mehrtens F Kranz H Bednarek P Weisshaar B 2005 The Arabidopsis transcription factor MYB12 is a flavonol-specific regulator of phenylpropanoid biosynthesis. Plant Physiology 138, 1083–1096 15923334 10.1104/pp.104.058032 PMC1150422

[CIT0045] Navarre DA Payyavula RS R S Knowles NR Pillai S 2013 Changes in potato phenylpropanoid metabolism during tuber development. Plant Physiology and Biochemistry 65, 89–101 23434926 10.1016/j.plaphy.2013.01.007

[CIT0046] Navarre DA Pillai S Shakya R Holden MJ 2011 HPLC profiling of phenolics in diverse potato genotypes. Food Chemistry 127, 34–41

[CIT0047] Neer EJ Schmidt CJ Nambudripad R Smith TF 1994 The ancient regulatory-protein family of WD-repeat proteins. Nature 371, 297–300 8090199 10.1038/371297a0

[CIT0048] Nesi N Debeaujon I Jond C Pelletier G Caboche M Lepiniec L 2000 The TT8 gene encodes a basic helix-loop-helix domain protein required for expression of DFR and BAN genes in *Arabidopsis* siliques. Plant Cell 12, 1863–1878 11041882 10.1105/tpc.12.10.1863 PMC149125

[CIT0049] Nicholas KB Nicholas HBJ Deerfield DWI 1997 GeneDoc: analysis and visualization of genetic variation. EMBNEW.NEWS 4, 14

[CIT0050] Ohta M Matsui K Hiratsu K Shinshi H Ohme-Takagi M 2001 Repression domains of class II ERF transcriptional repressors share an essential motif for active repression. Plant Cell 13, 1959–1968 11487705 10.1105/TPC.010127 PMC139139

[CIT0051] Pang Y Wenger JP Saathoff K 2009 A WD40 repeat protein from *Medicago truncatula* is necessary for tissue-specific anthocyanin and proanthocyanidin biosynthesis but not for trichome development. Plant Physiology 151, 1114–1129 19710231 10.1104/pp.109.144022 PMC2773055

[CIT0052] Parr AJ Bolwell GP 2000 Phenols in the plant and in man. The potential for possible nutritional enhancement of the diet by modifying the phenols content or profile. Journal of the Science of Food and Agriculture 80, 985–1012

[CIT0053] Pattanaik S Kong Q Zaitlin D Werkman JR Xie CH Patra B Yuan L 2010 Isolation and functional characterization of a floral tissue-specific R2R3 MYB regulator from tobacco. Planta 231, 1061–1076 20157728 10.1007/s00425-010-1108-y

[CIT0054] Payyavula RS Navarre DA Kuhl JC Pantoja A Pillai SS 2012 Differential effects of environment on potato phenylpropanoid and carotenoid expression. BMC Plant Biology 12, 39 22429339 10.1186/1471-2229-12-39 PMC3342224

[CIT0055] Prior RL 2003 Fruits and vegetables in the prevention of cellular oxidative damage. American Journal of Clinical Nutrition 78, 570S-–578S 12936951 10.1093/ajcn/78.3.570S

[CIT0056] Quattrocchio F Wing JF Va K De Woude Mol JNM Koes R 1998 Analysis of bHLH and MYB domain proteins: species-specific regulatory differences are caused by divergent evolution of target anthocyanin genes. The Plant Journal 13, 475–488 9680994 10.1046/j.1365-313x.1998.00046.x

[CIT0057] Reichert AI He XZ Dixon RA 2009 Phenylalanine ammonia-lyase (PAL) from tobacco (*Nicotiana tabacum*): characterization of the four tobacco PAL genes and active heterotetrameric enzymes. Biochemical Journal 242, 233–242 19725811 10.1042/BJ20090620

[CIT0058] Rommens CM Richael CM Yan H Navarre DA Ye J Krucker M Swords K 2008 Engineered native pathways for high kaempferol and caffeoylquinate production in potato. Plant Biotechnology Journal 6, 870–886 18662373 10.1111/j.1467-7652.2008.00362.x

[CIT0059] Rourke N O Hatcher L Stepanski EJ 2005 A step-by-step approach to using SAS for univariate and multivariate statistics , 2nd edition. Cary, NC: SAS Institute Inc

[CIT0060] Sablowski RW Moyano E Culianez-Macia FA Schuch W Martin C Bevan M 1994 A flower-specific Myb protein activates transcription of phenylpropanoid biosynthetic genes. EMBO Journal 1, 128–137 8306956 10.1002/j.1460-2075.1994.tb06242.x PMC394786

[CIT0061] Shakya R Navarre DA 2006 Rapid screening of ascorbic acid, glycoalkaloids, and phenolics in potato using high-performance liquid chromatography. Journal of Agricultural and Food Chemistry 54, 5253–5260 16848503 10.1021/jf0605300

[CIT0062] Singleton VL Rossi Jr JA 1965 Colorimetry of total phenolics with phosphomolybdic-phosphotungstic acid reagents. American Journal of Enology and Viticulture 16, 144–158

[CIT0063] Solanol R Nieto C Avila J Cafias L Diaz I Paz-Ares J 1995 Dual DNA binding specificity of a petal epidermis specific MYB transcription factor (MYB.Ph3) from *Petunia hybrida* . EMBO Journal 14, 1773–1784 7737128 10.1002/j.1460-2075.1995.tb07166.x PMC398271

[CIT0064] Solfanelli C Poggi A Loreti E Alpi A Perata P 2006 Sucrose-specific induction of the anthocyanin biosynthetic pathway in *Arabidopsis* . Plant Physiology 140, 637–646 16384906 10.1104/pp.105.072579 PMC1361330

[CIT0065] Stracke R Werber M Weisshaar B 2001 The R2R3-MYB gene family in *Arabidopsis thaliana* . Current Opinion in Plant Biology 4, 447–456 11597504 10.1016/s1369-5266(00)00199-0

[CIT0066] Stushnoff C Ducreux LJ Hancock RD 2010 Flavonoid profiling and transcriptome analysis reveals new gene-metabolite correlations in tubers of *Solanum tuberosum* L. Journal of Experimental Botany 61, 1225–1238 20110266 10.1093/jxb/erp394 PMC2826661

[CIT0067] Tamura K Dudley J Nei M Kumar S 2007 MEGA4: Molecular evolutionary genetics analysis (MEGA) software version 4.0. Molecular Biology and Evolution 24, 1596–1599 17488738 10.1093/molbev/msm092

[CIT0068] Teng S Keurentjes J Bentsink Ln Koornneef M Smeekens S 2005 Sucrose-specific induction of anthocyanin biosynthesis in *Arabidopsis* requires the MYB75/PAP1 gene. Plant Physiology 139, 1840–1852 16299184 10.1104/pp.105.066688 PMC1310563

[CIT0069] **The Potato Genome Sequencing Consortium** 2011 Genome sequence and analysis of the tuber crop potato. Nature 475, 189–195 21743474 10.1038/nature10158

[CIT0070] Verhaak RG Sanders MA Bijl MA Delwel R Horsman S Moorhouse MJ van der Spek PJ Lowenberg B Valk PJ 2006 HeatMapper: powerful combined visualization of gene expression profile correlations, genotypes, phenotypes and sample characteristics. BMC Bioinformatics 7, 337 16836741 10.1186/1471-2105-7-337 PMC1574351

[CIT0071] Vetten Nd F Quattrocchio Mol J Koes R 1997 The *an11* locus controlling flower pigmentation in petunia encodes a novel WD-repeat protein conserved in yeast, plants, and animals. Genes & Development 11, 1422–1434 9192870 10.1101/gad.11.11.1422

[CIT0072] Vinson JA Demkosky CA Navarre DA Smyda MA 2012 High-antioxidant potatoes: acute in vivo antioxidant source and hypotensive agent in humans after supplementation to hypertensive subjects. Journal of Agricultural and Food Chemistry 60, 6749–6754 10.1021/jf2045262 22224463

[CIT0073] Vogt T 2010 Phenylpropanoid biosynthesis. Molecular Plant 3, 2–20 20035037 10.1093/mp/ssp106

[CIT0074] Voinnet O Rivas S Mestre P Baulcombe D 2003 An enhanced transient expression system in plants based on suppression of gene silencing by the p19 protein of tomato bushy stunt virus. The Plant Journal 33, 949–956 12609035 10.1046/j.1365-313x.2003.01676.x

[CIT0075] Walker AR Davison PA Bolognesi-Winfield AC James CM Srinivasan N Blundell TL Esch JJ Marks MD Gray JC 1999 The *TRANSPARENT TESTA GLABRA1* locus, which regulates trichome differentiation and anthocyanin biosynthesis in *Arabidopsis*, encodes a WD40 repeat protein. Plant Cell 11, 1337–1349 10402433 10.1105/tpc.11.7.1337 PMC144274

[CIT0076] Wrolstad RE Durst RW Lee J 2005 Determination of total monomeric anthocyanin pigment content of fruit juices, beverages, natural colorants, and wines by the pH differential method: Collaborative study. Journal of AOAC International 88, 1269–1278 16385975

[CIT0077] Wu X Beecher GR Holden JM Haytowitz DB Gebhardt SE Prior RL 2006 Concentrations of anthocyanins in common foods in the United States and estimation of normal consumption. Journal of Agricultural and Food Chemistry 54, 4069–4075 16719536 10.1021/jf060300l

[CIT0078] Yanhui C Xiaoyuan Y Kun H 2006 The MYB transcription factor superfamily of *Arabidopsis*: expression analysis and phylogenetic comparison with the rice MYB family. Plant Molecular Biology 60, 107–124 16463103 10.1007/s11103-005-2910-y

[CIT0079] Zhang Y Cheng S De Jong D Griffiths H Halitschke R De Jong W 2009a The potato *R* locus codes for dihydroflavonol 4-reductase. Theoretical and Applied Genetics 119, 931–937 19588118 10.1007/s00122-009-1100-8 PMC2729421

[CIT0080] Zhang Y Jung CS De Jong WS 2009b Genetic analysis of pigmented tuber flesh in potato. Theoretical and Applied Genetics 119, 143–150 19363602 10.1007/s00122-009-1024-3 PMC2690854

[CIT0081] Zimmermann IM Heim MA Weisshaar B Uhrig JF 2004 Comprehensive identification of *Arabidopsis thaliana* MYB transcription factors interacting with R/B-like BHLH proteins. Plant Journal 40, 22–34 15361138 10.1111/j.1365-313X.2004.02183.x

[CIT0082] Zucker M Levy CC 1959 Some factors which affect the synthesis of chlorogenic acid in disks of potato tuber. Plant Physiology 34, 108–112 16655184 10.1104/pp.34.2.108 PMC541155

[CIT0083] Zucker M 1965 Induction of phenylalanine deaminase by light and its relation to chlorogenic acid synthesis in potato tuber tissue. Plant Physiology 40, 779–784 16656157 10.1104/pp.40.5.779 PMC550380

