# Supplementary material for: Transcription factors, sucrose, and sucrose metabolic genes interact to regulate potato phenylpropanoid metabolism
Source: J Exp Bot. 2013 Oct 5;64(16):5115–31. doi: 10.1093/jxb/ert303 (PMC3830490; doi:10.1093/jxb/ert303)
Supplement: Supplementary Data [file supp_64_16_5115__index.html]

Transcription factors, sucrose, and sucrose metabolic genes interact to regulate potato phenylpropanoid metabolism — Transcription factors, sucrose, and sucrose metabolic genes interact to regulate potato phenylpropanoid metabolism — Supplementary Data 

# Transcription factors, sucrose, and sucrose metabolic genes interact to regulate potato phenylpropanoid metabolism

## Supplementary Data

Data files

**Files in this Data Supplement:**

- Supplementary Data - Supplementary Data
