# Supplementary material for: Transcription factors, sucrose, and sucrose metabolic genes interact to regulate potato phenylpropanoid metabolism
Source: J Exp Bot. 2013 Oct 5;64(16):5115–31. doi: 10.1093/jxb/ert303 (PMC3830490; doi:10.1093/jxb/ert303)
Supplement: Supplementary Data [file supp_ert303_jexbot097709_file001.pdf]

Supplementary data

Transcription factors, sucrose and sucrose metabolic genes interact to regulate potato phenylpropanoid metabolism

Raja S Payyavula, Rajesh Singh, and Duroy A Navarre*

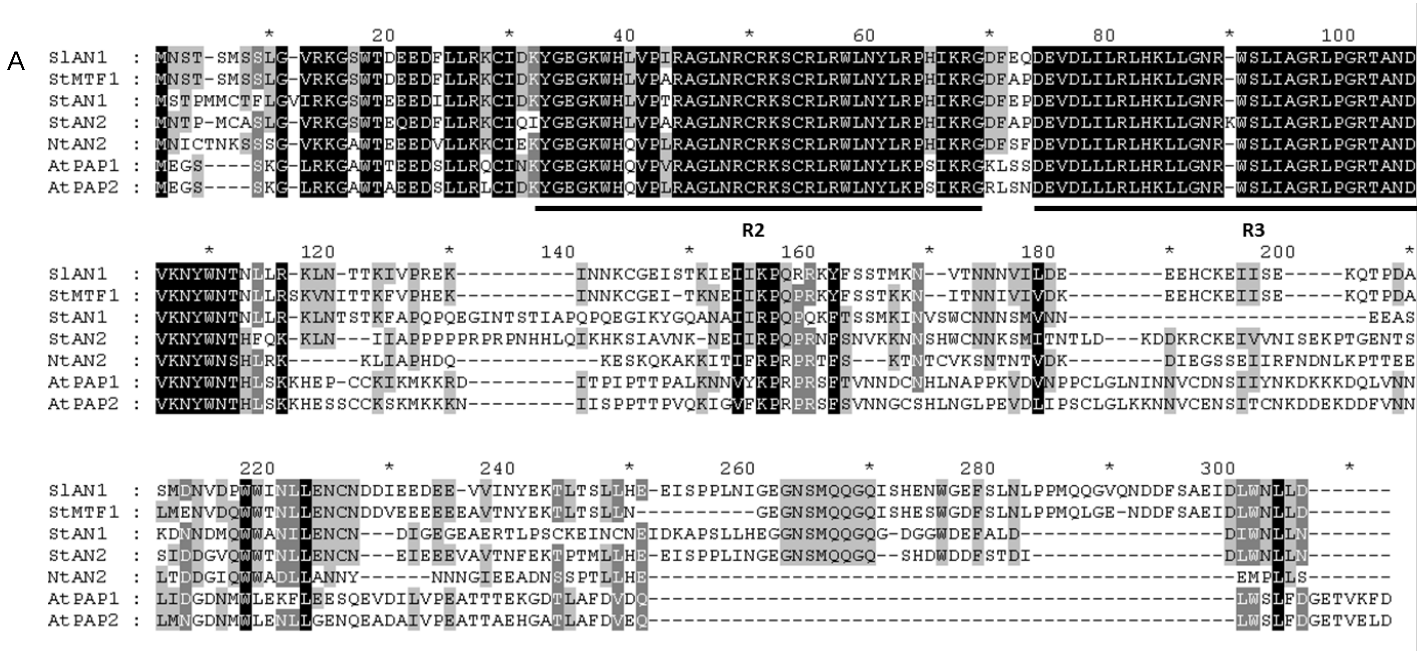

Supplementary Fig. S1.

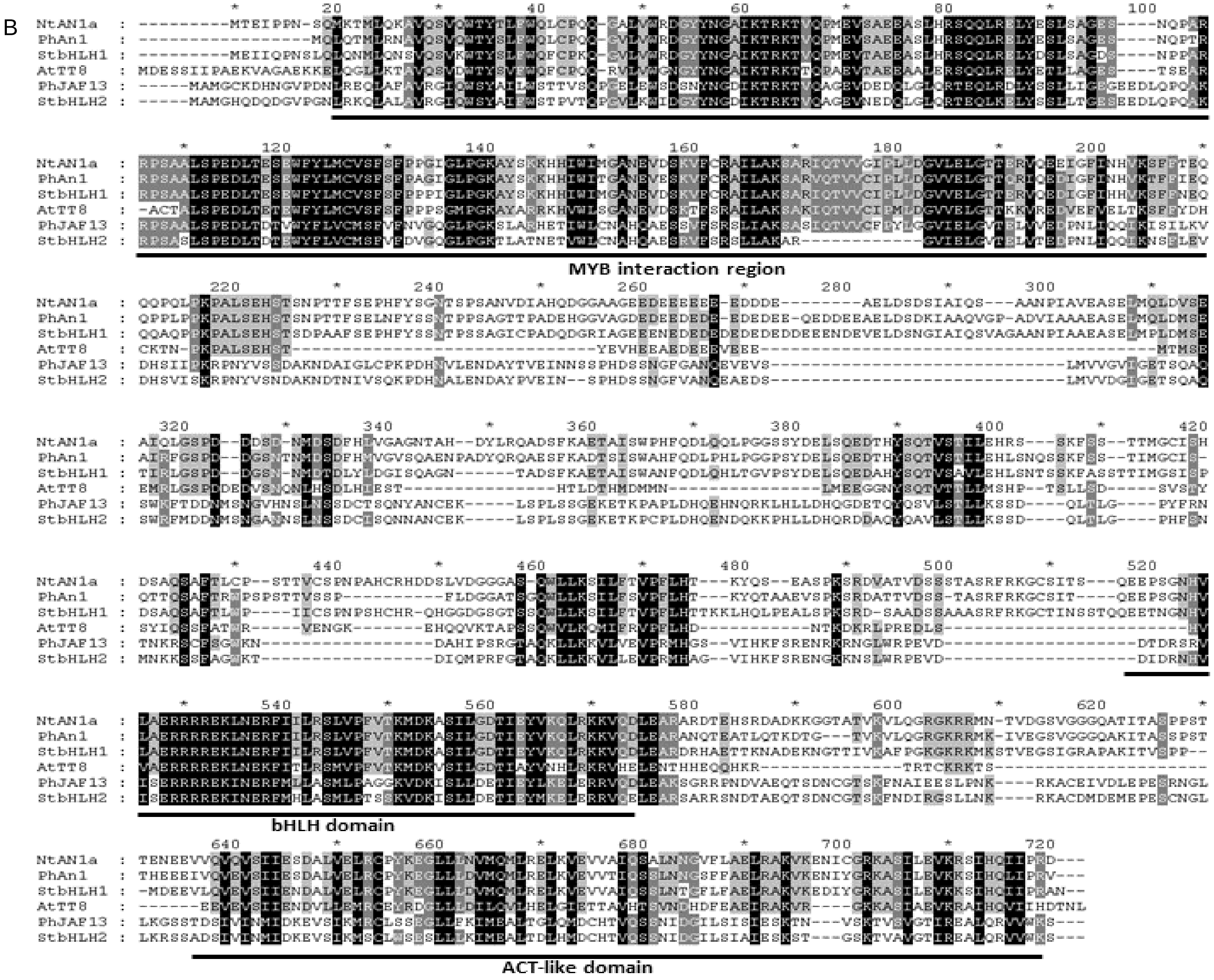

Supplementary Fig. S1 continued.

C

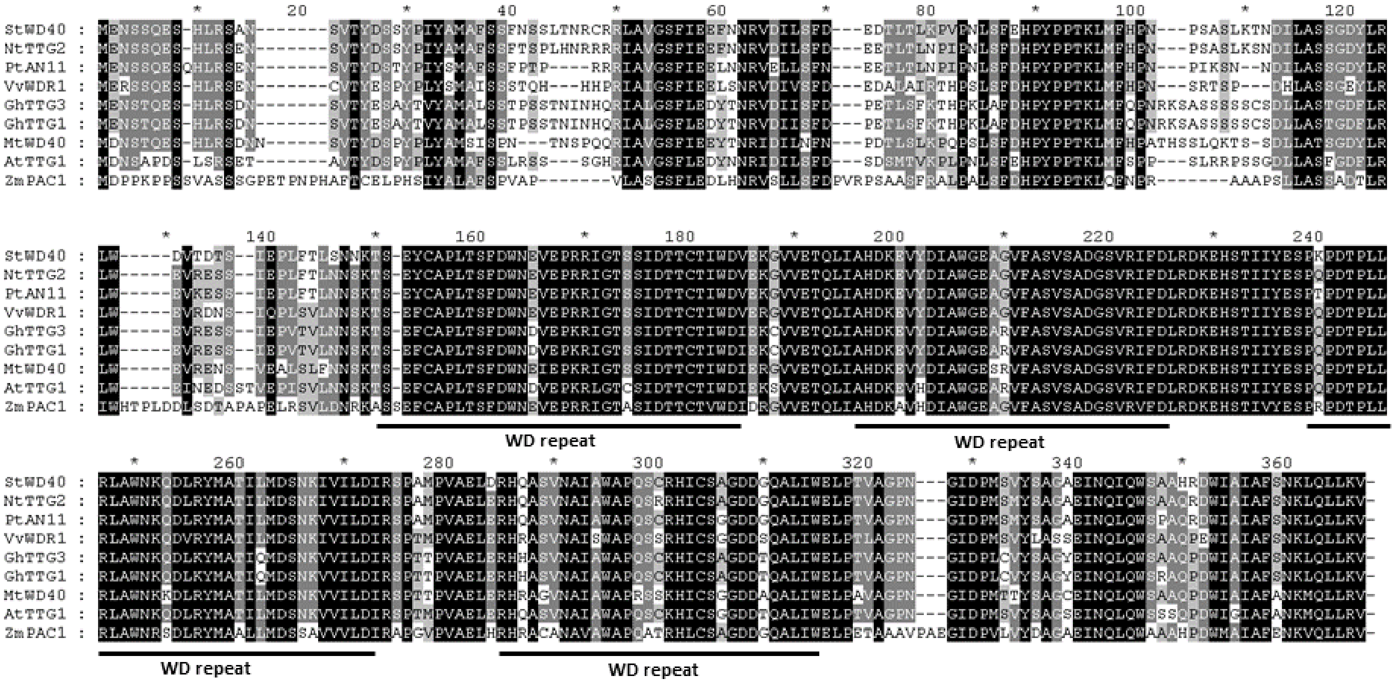

D

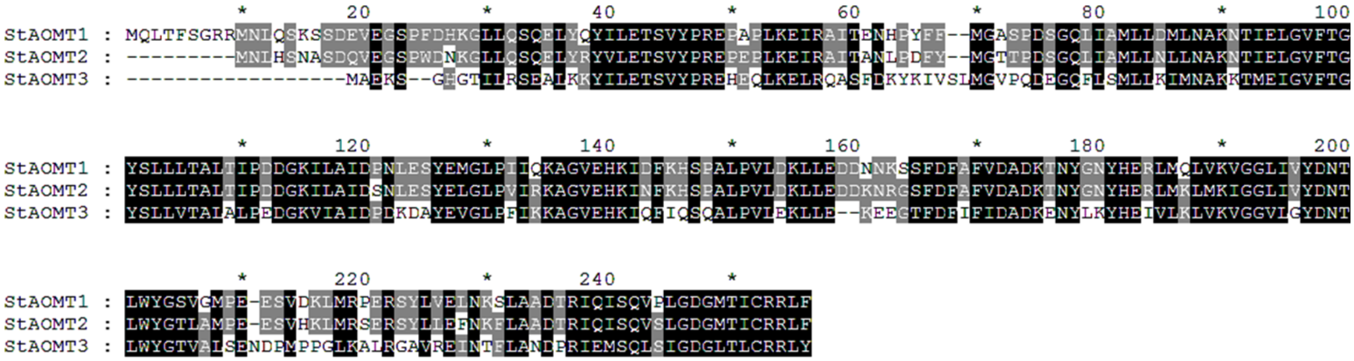

Supplementary Fig. S1 continued. Protein sequence alignment of representative (A) MYBs, (B) bHLH, (C) WD40 and (D) AOMT from potato and from other species. Accession numbers are listed in Fig. 2 or Supplementary Table 3.

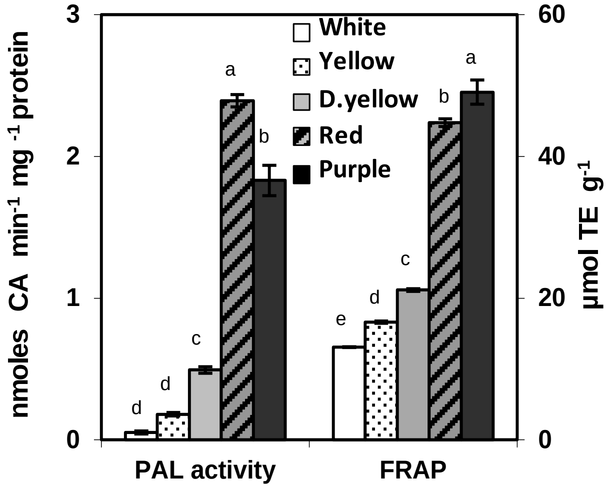

Supplementary Fig. S2. PAL enzyme activity and FRAP antioxidant activity in the five genotypes. The data represents the means ± SE of three biological replicates. Values with same letter are not significantly different ($p < 0.05$).

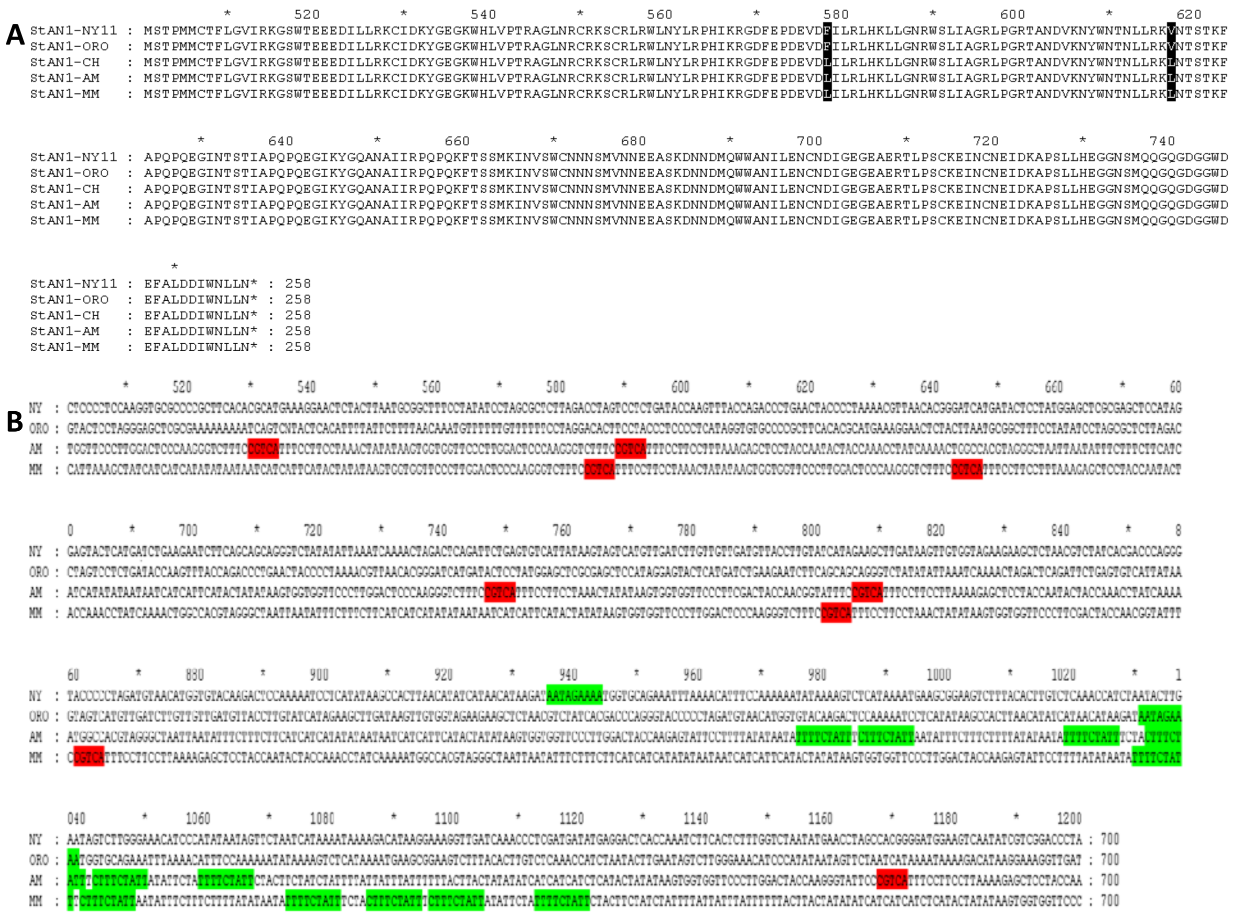

Supplementary Fig. S3. (A) StAN1 protein sequence of the five potato genotypes used in this study. Differences are shaded in black. Coding regions were sequenced and translated. (B) *StAN1* promoter region from four genotypes used in this study. 1500 bp were sequenced and a 700 bp region containing the SURE and jasmonate motifs starting 500 bp upstream of the start codon were aligned as shown. Methyl jasmonate responsive motifs are highlighted in red. Sugar responsive elements (SURE) are highlighted in green.

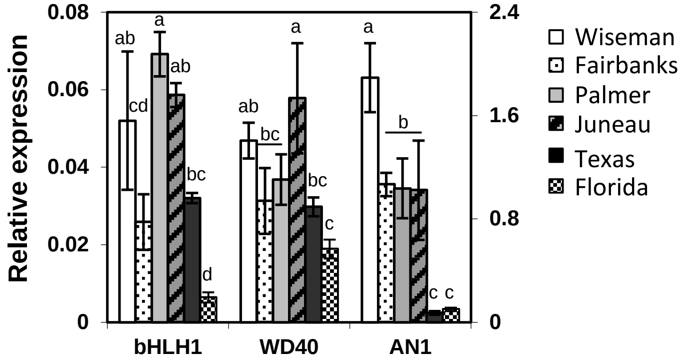

Supplementary Fig. S4. Expression of selected transcription factors in purple potatoes grown in in Alaska (Wiseman, Fairbanks, Palmer and Juneau), Texas and Florida. *AN1* expression uses the y-axis on the right. The data represents the means ± SE of three biological replicates.

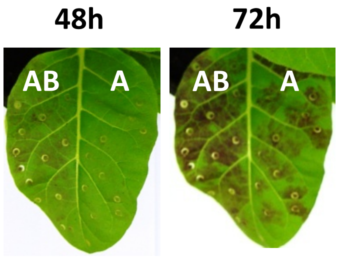

Supplementary Fig. S5. Tobacco leaves 48h and 72h after infiltrating with a binary construct harboring *AN1* (A) alone or *AN1* + *bHLH1* (AB).

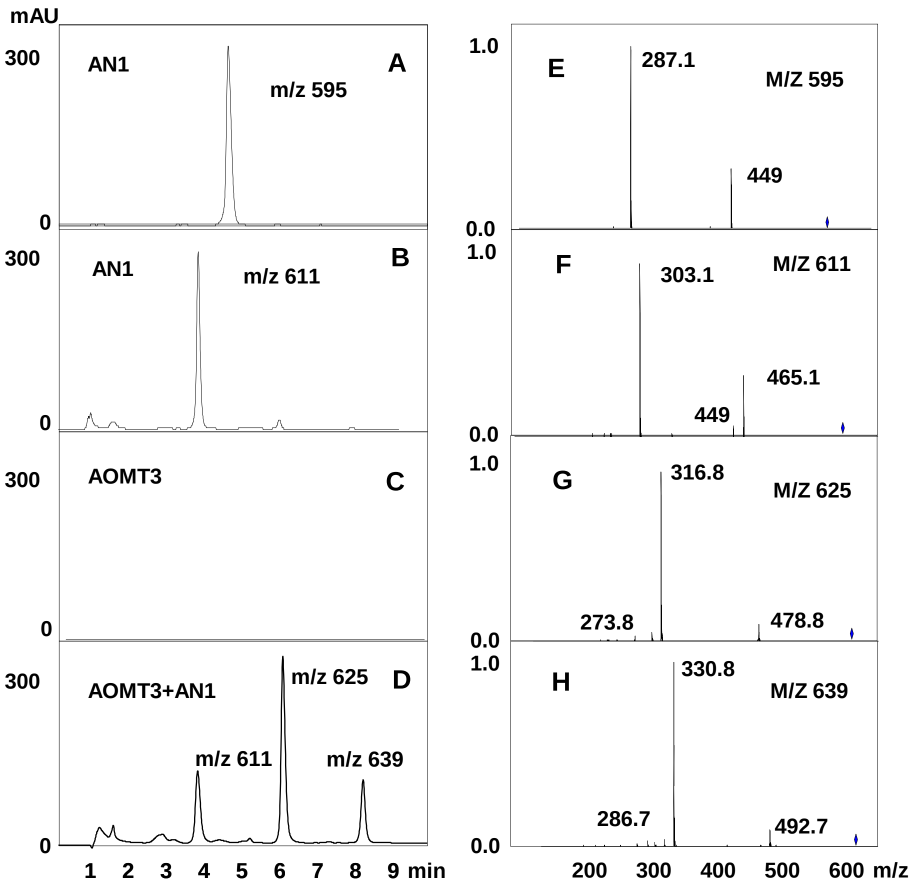

Supplementary Fig. S6. MS extracted ion data of the most abundant anthocyanins formed in (A) *N. tabacum* (Samsun) infiltrated with *AN1*; (B) *N. benthamiana* leaves infiltrated with *AN1*, (C) *AOMT3* and (D) *AN1+AOMT3*. MS^2 data for the indicated peaks is shown in panels E-H.

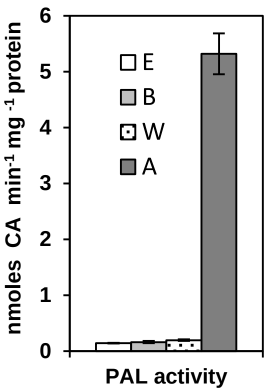

Supplementary Fig. S7. PAL activity in tobacco leaves infiltrated with empty vector (E), *bHLH1* (B), *WD40* (W) or *AN1* (A). The data represents the mean ± SE of three biological replicates.

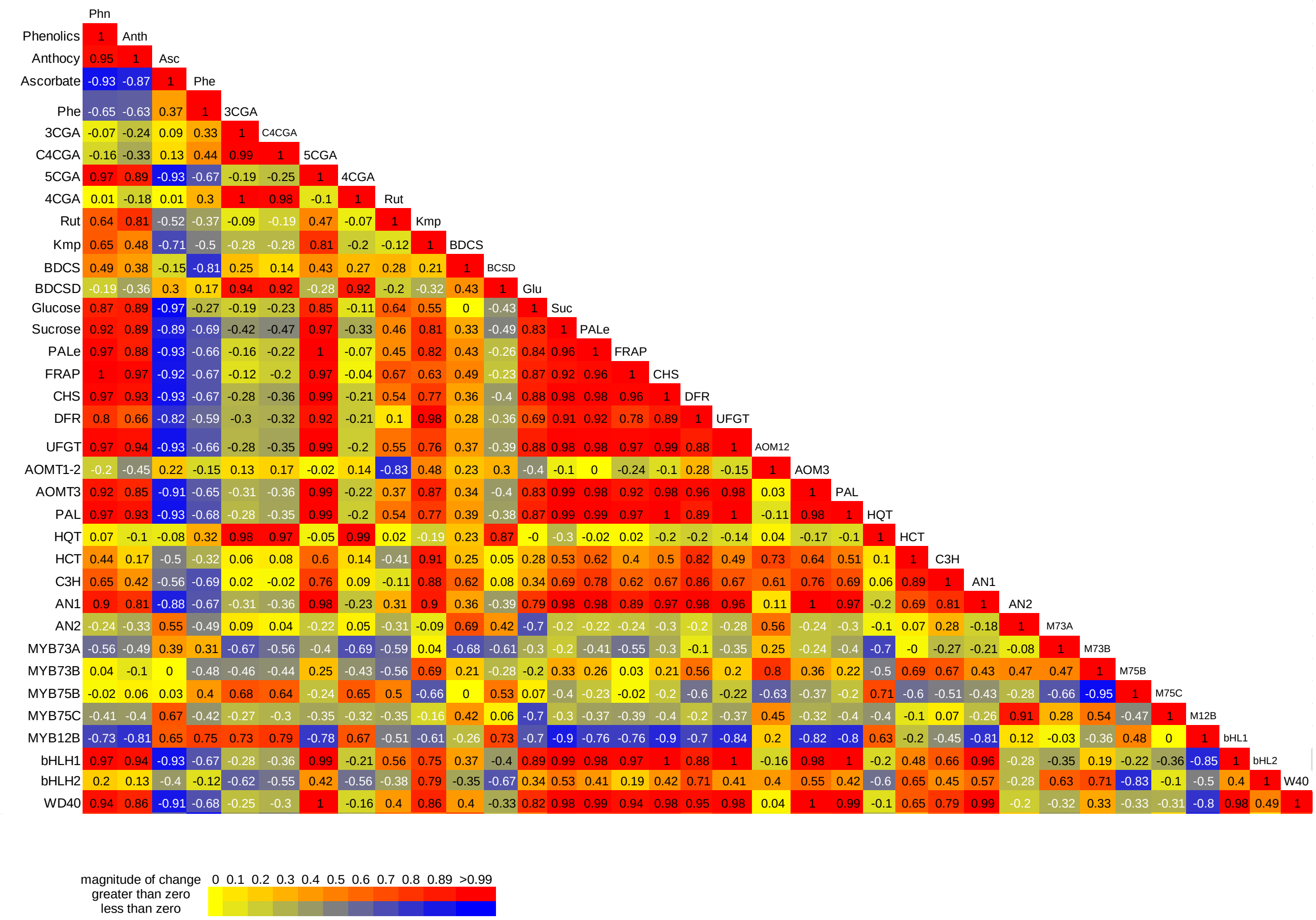

Supplementary Fig. S8. Correlation analysis of transcript and metabolite expression in tubers from five potato genotypes. Pearson correlation coefficients are shown.

Supplementary Table S1. Retention times and MS data of select compounds present in potato extracts separated by HPLC.

RT	Abbreviation	Compound	*m/z*	MS/MS (*m/z*)
1.8	Asc	ascorbic acid	175 (M-H)	115, 87, 71
2.5	Tyr	tyrosine	180 (M-H)	163, 119, 93
4.0	Phe	phenylalanine	164 (M-H)	147, 148, 72
5.9	Trp	tryptophan	203 (M-H)	159, 116, 142, 186
6.5	3CGA	3-*o*-chlorogenic acid	353 (M-H)	191, 179, 135
7.0	CP	caffeoyl putrescine	251 (M+H)	234, 163, 89, 115
7.6	C4CGA	cis-4-chlorogenic acid	353 (M-H)	173, 179, 135, 191
8.3	5CGA	5-*o*-chlorogenic acid	353 (M-H)	191,179, 161,135
8.4	BDCS	bis-dihydrocaffeoyl spermine	531 (M+H)	293, 222, 513, 165, 367
8.5	4CGA	4-*o*-chlorogenic acid	353 (M-H)	173, 179, 191, 135
9.1	C5CGA	cis-5-chlorogenic acid	353 (M-H)	191, 179, 173
9.6	BDCSD	bis-dihydrocaffeoyl spermidine	474 (M+H)	222, 457, 165, 310, 236
10.4	FQA1	feruloyl quinic acid1	367 (M-H)	161, 135, 335
11.5	FQA2	feruloyl quinic acid2	367 (M-H)	179, 135, 191, 161, 143
12.0	TDCS	tris-dihydrocaffeoyl spermine	695 (M+H)	293, 222, 531, 474, 457
12.3	QRG	quercetin rutinoside-glucoside	771 (M-H)	591,300, 301, 609
12.6	QDG	quercetin diglucoside	625 (M-H)	300, 301, 445 607, 463
12.8	Myr	myricetin rutinoside	625 (M-H)	317, 316, 287, 607,461, 271
13.4	KRG	kaempferol rutinoside-glucoside	755 (M-H)	575, 285, 593
13.5	TDCSD	tris-dihydrocaffeoyl spermidine	638 (M+H)	456, 474, 293, 222
14.0	Rut	quercetin 3-rutinoside	609 (M-H)	301, 609, 271, 343
14.6	Kmp	kaempferol 3-rutinoside	593 (M-H)	285, 593, 431
14.8	RR	rhamnetin rutinoside	623 (M-H)	315, 300, 316, 271
15.4	Glyc	solanine	852 (M+H)	706, 398, 560
15.4	Glyc	chaconine	868 (M+H)	398, 706, 722, 560, 380

Supplementary Table S2. Retention times and MS data of anthocyanins in potato phenolic extracts separated by HPLC.

RT	Abbreviation	Compound	$[M]^+$ m/z	MS/MS (m/z)
3.5	PtRG	petunidin 3-rutinoside-5-glucoside	787	625,317,479
3.8	PlRG	pelargonidin 3-rutinoside-5-glucoside	741	579,271,433
3.9	DR	delphinidin 3-rutinoside	611	303, 465, 449
4.6	DRG	delphinidin 3-rutinoside-glucoside	773	303,611,465
5	CR	cynanidin 3-rutinoside	595	287,449
5.2	MRG	malvidin 3-rutinoside-5-glucoside	801	639,331,493
5.4	PlG	pelargonidin 3-glucoside	433	271
6.1	PlDGG	pelargonidin 3-diglucoside-5-glucoside	757	271,595
6.2	PeR	pelargonidin 3-rutinoside	579	271,433
6.3	PtR	petunidin 3-rutinoside	625	317,479
6.5	PlR	pelargonidin 3-rutinoside	579	271,433
7.4	PoCG	peonidin 3-(coumaroyl)-glucoside	609	301,463
8.2	MR	malvidin 3-rutinoside	639	331, 493
8.8	DCRG	delphinidin-3-(coumaroyl) rutinoside-5-glucoside	919	303,757,465
8.9	PlCRG	pelargonidin 3-(coumaryol) rutinoside-5-glucoside	887	725,271,433
9.06	PtCfRG	petunidin 3-(caffeoyl) rutinoside-5-glucoside	949	787,317,479
9.3	PlCRG	pelargonidin 3-(coumaroyl) rutinoside-5-glucoside	887	725,271,433
9.7	PeCRG	peonidin 3-(coumaroyl) rutinoside-5-glucoside	917	301,755,463
9.8	PlCDG	pelargonidin 3-(coumaroyl) diglucoside-5-glucoside	903	741,271,433
10.1	CCRG	cyanidin 3-(coumaroyl) rutinoside-5-glucoside	903	741,287,449
10.3	PtCRG	petunidin 3-(coumaroyl) rutinoside-5-glucoside	933	317,771,479
11	PlCRG	pelargonidin 3-(coumaroyl) rutinoside-5-glucoside	887	725,271,433
11.1	PtFRG	petunidin 3-(feruloyl) rutinoside-5-glucoside	963	317,801,479
11.4	PeCRG	peonidin 3-(coumaroyl) rutinoside-5-glucoside	917	755,301,463
11.8	MCRG	malvidin 3-(coumaroyl) rutinoside-5-glucoside	947	785,331,493
12	PlFRG	pelargonidin 3-(feruloyl) rutinoside-5-glucoside	917	755,271,433
12.3	PtCR	petunidin 3-(coumaroyl)-rutinoside	771	317,479,245
12.5	MFRG	malvidin 3-(feruloyl) rutinoside-5-glucoside	977	331,815,493
13.3	PlRG	pelargonidin rutinoside-5-glucoside	725	271,433
13.7	PeRG	peonidin rutinoside-5-glucoside	755	301,463

Supplementary Table S3. List of primers used in this study.

Species	Primer	Primer sequence	Used for	Accession no or Potato Genome Contig
Potato	StMYB73A-F	GCCAGACAAGGTATTTGTGCCGTT	QPCR	PGSC0003DMS000002386
	StMYB73A-R	TGCGATTAGCAGCTCTATCCCTGA		
	StMYB73B-F	GCCGGTGGTTGATAAACCGATTCC		PGSC0003DMS000003180
	StMYB73B-R	GCATTCCTTATTGCATCRGTCTGC		
	StMYB12A-F	TGTTTATGGGACGGAGAAGGAG		PGSC0003DMS000001330
	StMYB12A-R	GCCAAGTTACCAAATCACTCTGCATGG		
	StMYB12B-F	AGGAGATTATGCCAGACGTGGTGA		PGSC0003DMS000000077
	StMYB12B-R	CATGGTGGCGTTGGCCATACTAAT		
	StAN1-F	AAGTATGGGCAAGCCAATGCCA		PGSC0003DMS000000121
	StAN1-R	GCCCACCATTGCATATCGTTGTTGTC		
	StAN2-F	AGACCTCAACCTCGGAACTTCTCA		PGSC0003DMS000000777
	StAN2-R	GTCCACCATTGAACTCCATCGTCT		
	StMYB75A-F	CACTCCTCAAGTTGTGTTTGTCCC		PGSC0003DMS000000511
	StMYB75A-R	TGAAGCTATGTTGCTCGGGCTCTT		
	StMYB75B-F	TGCACCAAAGCCCATAAGGATTTC		PGSC0003DMT400031607
	StMYB75B-R	GCAGCTTTCTCCCATAAGAATTTCA		
	StMYB75C-F	TGGTCCCTCATTGCCGGAAGAATA		PGSC0003DMT400085829
	StMYB75C-R	GCGGCTCATTTCCTTGTCCTTTGA		
	StMTF1-F	AGCAAACTCCAGATGCATTGATGG		EU310399
	StMTF1-R	CCTTGTTGCATGGAGTTACCTTCACC		
	StMTF2-F	TGCTGAAAGCAAAGGTGGAATGGG		PGSC0003DMS000000013
	StMTF2-R	TAATAGCCTCAGTTTCAGCCTCAGGG		
	StMYB10-F	GCATCAAGATTGCCAGGAAGAACAG		PGSC0003DMT400060168
	StMYB10-R	AGTGGATGGTAGATCATTGTGTTGT		
	StWD40-F	ATTCAAGTCAAGAATCGCATCTCCG		PGSC0003DMT400001513
	StWD40-R	GAACTCTTCGATAAAGCTYCCGAC		
	StbHLH1-F2	TCTCTTGGAYGGTGTAGTGGAACT		PGSC0003DMT400033569
	StbHLH1-R2	AACAGCGGATGACGGAGTATTGCT		
	StbHLH2-F2	AGGCSAGATCAGCAAGACGATCAA		PGSC0003DMT400032139
	StbHLH2-R2	AGCTCTCGCTCCAAAGACAACTCA		
	StAOMT-1/2F	CCTGTTCTTGACAAACTCCTGGAAG		PGSC0003DMT400006134 and
	StAOMT-1/2R	TCAYCAGCTKCATTAGTCTCTCGT		PGSC0003DMT400006136
	StAOMT-3F	GGTGAAAGTTGGAGGAGTACTTGGCT		PGSC0003DMT400016512
	StAOMT-3R	TGCCTTTAATCCTGGTGGCATAGG		
	StAN1-F-FL	AATGCGGCCGCATGAGTACTCCTATGATGTGTAC	Cloning	
	StAN1-R-FL	CCGGGTACCTTAATTAAGTAGATTCCATATATC		
	StbHLH1-F-FL	AATGCGGCCGCATGGAGATTATACAGCCTAATAGC		
	StbHLH1-R-FL	CCGGGTACCTTAATTAGCTCTAGGGATTATCTG		
	StWD40-F-FL	AATGCGGCCGCATGGARAATTCAAGTCAAGAATCG		
	StWD40-R-FL	CCGGGTACCTTATACTTTAAGCAGCTGCAACTTG		
Vector	35S-F	CAAGACCCTTCCTCTATAT		
	pORE-F	ACTGAAGGCGGGAAACGACAATCT		
	pORE-R	AGCGGATAACAATTTCACACAGG		

Supplementary Table S3 continued.

Species	Primer	Primer sequence	Used for	Accession no
Tobacco	NtINV1-F	TTAGAGACCCAACGACTGCTTGGA	QPCR	AJ305044
	NtINV1-R	ACCCGTTTGCCTCATCGGTTGATA		
	NtSUSY-F	TTCACCTCAYGGATATTTCGCCCA		AB055497
	NtSUSY-R	AGTCCTTGCTCCTTTATGCGCT		
	NtCHS-F	ACTTGGYAAAGAGGCWGCCCAA		AF311783
	NtCHS-R	CGGCAAAGCAACCTTGTTGGTA		
	NtPAL1/4-F	TTCGCCTACGCTGATGATCCTT		M84466/EU883669
	NtPAL1/4-R	TGCAGCTCTTGCACTCTCAACT		
	NtPAL2/3-F	ACCCACTGATGCAGAAACTAAGGC		D17467/X78269
	NtPAL2/3-R	GCAATTGCAGGGTTCCCAYTTT		
	NtDFR-F	ATTCATCTGCGCRTCCCATCAT		AB289448
	NtDFR-R	AAATACACCACRGGCAAGTCCT		
	NtF3H-F	AGGCYTTAACCAAGGCATGTGT		AB289450
	NtF3H-R	CGGGCTGAACAGTRATCCAAGT		
	NtBHLH1-F	ATGKGCGCAAACGAGGTTGATAGC		HQ589208/HQ589209
	NtBHLH1-R	TRGCTGAGGTTGTTGTTGCTCA		
	NtINV1-F	TTAGAGACCCAACGACTGCTTGGA		AJ305044
	NtINV1-R	ACCCGTTTGCCTCATCGGTTGATA		
	NtINV2-F	TATACCGGGTCAACCAACGAGTCA		HM022269
	NtINV2-R	CATTTGCCTTGTGGTGTGGTCCAT		
	NtActin-F	AAGCTGTGTTGTCCCTATACGCCA		AB158612
	NtActin-R	ATCGCGGACAATTTCCCGTTCA		
	NtELF-F	TGAACCATCCAKGACAGATTGG		D63396
	NtELF-R	TGGGCTCCTTCTCAATCTCCTT		

Supplementary Table 4. Protein similarity matrix of transcription factors from different species. (A) MYB (B) bHLH and (W) WD40. Accession numbers are listed in Fig. 2.

A

	StMTF1	StAN1	StAN2	NtAN2	AtPAP1	StMYB75A
SlAN1	89%	64%	70%	57%	56%	45%
StMTF1		64%	71%	58%	55%	45%
StAN1			66%	58%	55%	45%
StAN2				61%	53%	48%
NtAN2					56%	43%
AtPAP1						43%

B

	PhAn1	StbHLH1	AtTT8	PhJAF13	StbHLH2
NtAN1a	83%	81%	54%	43%	44%
PhAn1		80%	53%	43%	44%
StbHLH1			53%	43%	43%
AtTT8				42%	42%
PhJAF13					86%

C

	NtTTG2	PhAN11	VvWDR1	MtWD40	AtTTG1
StWD40	97%	94%	88%	87%	88%
NtTTG2		95%	89%	88%	88%
PhAN11			90%	88%	88%
VvWDR1				89%	88%
MtWD40					87%

Supplementary Table S5. List of regulatory elements in promoters of different genes from *S. tuberosum* group Phureja. The numbers in each column represent the position of the element in the promoter sequence relative to the ATG start codon.

	Promoter elements (sequence)			
**Genes**	**MYBCORE (CNGTTR)**	**MYBPLANT (MACCWAMC)**	**MYCCONSENSUS (CANNTG)**	**SURE (AATAGAAAA)**
**AN1**	-1380, -1279, -786	none	-1349, -13	-604, -561, -521
**bHLH1**	-737, -703	-949	-1466, -1154, -960, -510, -409	none
**WD40**	-1422, -1132, -1096, -908, -678, -437, -133	-63	-1150, -1123, -956, -711	-214
**PAL1**	-825, -271, -268	-1404, -337	-1385, -1346, -189	none
**HQT**	-1021, -569	none	-1299	-1082
**C3H**	-1368, -1329, -1217, -1079, -1067, -576	none	-1271, -1238, -1211, -1067, -1040, -614, -576, -526	none
**HCT**	-92	none	-872, -656, -597	-178
**CHS**	-366	none	-1336, -1171, -200, -178	none
**F3H**	-1082	none	-477, -335, -137	none
**DFR**	-1395, -1207, -1106, -1039, -874	-189	-1236, -1135, -884, -813, -561, -403, -331, -160, -46	none
**ANS**	-1330, -1203, -747, -628, -625	-386, -171	-747, -617, -521, -344, -227	none
**SUSY1**	-1396, -1365, -573, -541, -445, -51	-1299, -1086, -645	-1257, -1067, -998, -846, -730, -685, -7	-1279, -666
**SUSY4**	-1012, -271, -240, -202	-848	-1289, -1132, -650, -444, -240, -214, -202	none
**INV1**	none	-1442, -203	-1396, -1373, -1339, -1142, -1130, -1079, -1049	none
**INV2**	-717, -548, -83	-539, -476, -259, -198	-980, -622, -124, -83	none
